# Nitric oxide and nitrous oxide turnover in natural and engineered microbial communities: biological pathways, chemical reactions, and novel technologies

**DOI:** 10.3389/fmicb.2012.00372

**Published:** 2012-10-23

**Authors:** Frank Schreiber, Pascal Wunderlin, Kai M. Udert, George F. Wells

**Affiliations:** ^1^Department of Environmental Microbiology, Eawag - Swiss Federal Institute of Aquatic Science and TechnologyDübendorf, Switzerland; ^2^Department of Environmental Systems Sciences, Eidgenössische Technische HochschuleZurich, Switzerland; ^3^Department of Process Engineering, Eawag - Swiss Federal Institute of Aquatic Science and TechnologyDübendorf, Switzerland; ^4^Department of Civil, Environmental and Geomatic Engineering, Eidgenössische Technische HochschuleZurich, Switzerland

**Keywords:** isotopic signature, microsensors, molecular tools, dinitrogen oxide, nitrogen monoxide, pathway identification, quantum cascade laser absorption spectroscopy (QCLAS), site preference

## Abstract

Nitrous oxide (N_2_O) is an environmentally important atmospheric trace gas because it is an effective greenhouse gas and it leads to ozone depletion through photo-chemical nitric oxide (NO) production in the stratosphere. Mitigating its steady increase in atmospheric concentration requires an understanding of the mechanisms that lead to its formation in natural and engineered microbial communities. N_2_O is formed biologically from the oxidation of hydroxylamine (NH_2_OH) or the reduction of nitrite (NO^−^_2_) to NO and further to N_2_O. Our review of the biological pathways for N_2_O production shows that apparently all organisms and pathways known to be involved in the catabolic branch of microbial N-cycle have the potential to catalyze the reduction of NO^−^_2_ to NO and the further reduction of NO to N_2_O, while N_2_O formation from NH_2_OH is only performed by ammonia oxidizing bacteria (AOB). In addition to biological pathways, we review important chemical reactions that can lead to NO and N_2_O formation due to the reactivity of NO^−^_2_, NH_2_OH, and nitroxyl (HNO). Moreover, biological N_2_O formation is highly dynamic in response to N-imbalance imposed on a system. Thus, understanding NO formation and capturing the dynamics of NO and N_2_O build-up are key to understand mechanisms of N_2_O release. Here, we discuss novel technologies that allow experiments on NO and N_2_O formation at high temporal resolution, namely NO and N_2_O microelectrodes and the dynamic analysis of the isotopic signature of N_2_O with quantum cascade laser absorption spectroscopy (QCLAS). In addition, we introduce other techniques that use the isotopic composition of N_2_O to distinguish production pathways and findings that were made with emerging molecular techniques in complex environments. Finally, we discuss how a combination of the presented tools might help to address important open questions on pathways and controls of nitrogen flow through complex microbial communities that eventually lead to N_2_O build-up.

## Introduction

Nitric oxide (NO) and nitrous oxide (N_2_O) are atmospheric trace gases that influence atmospheric chemistry and the greenhouse effect. Biological and chemical processes produce N_2_O on the earth surface (Crutzen, 1979). Entering the stratosphere, N_2_O is converted to NO by photo-oxidation. NO together with nitrogen dioxide (NO_2_) participate in a set of reactions that transfer ozone (O_3_) to molecular oxygen (O_2_), thereby leading to O_3_ layer depletion. In fact, N_2_O is and will remain the dominant O_3_-depleting substance in the twenty-first century (Ravishankara et al., 2009), since the use of chlorofluorocarbons has been restricted by the Montreal Protocol. In addition, N_2_O is a potent greenhouse gas. The infrared radiative forcing of one N_2_O molecule is 206 times that of one carbon dioxide (CO_2_) molecule (Stein and Yung, 2003). Together with the long atmospheric lifetime of N_2_O (~120 years) this results in a ~300 times higher global warming potential of N_2_O than that of CO_2_ on a per molecule basis. Overall, N_2_O contributes 6–8% to the anthropogenic greenhouse effect, despite its relatively low atmospheric concentration (~322 ppbv) (Montzka et al., 2011).

Over the last 100 years atmospheric N_2_O concentrations have been steadily increasing due to the massive introduction of fixed nitrogen into the environment by humans (IPCC, 2001). Counteracting the further increase of N_2_O in the atmosphere will rely on (1) decreasing the introduction of fixed nitrogen into the environment by humans, (2) exactly quantifying the important environmental sources of N_2_O, and (3) implementing effective strategies to mitigate its formation in nitrogen-transforming, man-made ecosystems such as agriculture and wastewater treatment. Thus, there is an urgent need to understand the mechanisms that underpin the formation of N_2_O in natural and engineered microbial communities.

In this review, we will outline the current state-of-the-art on biological and chemical processes that can produce and consume N_2_O and NO—an important precursor of N_2_O in many biological pathways. We will discuss pathways that produce NO and N_2_O in natural and engineered microbial communities and experimental approaches that can be used to distinguish between different pathways in these systems. Importantly, NO and N_2_O formation can be highly dynamic and occur at small spatial scales. Thus, we will further introduce two novel technologies that provide such data and how they can lead to mechanistic insight: (1) NO and N_2_O microelectrodes and (2) the analysis of the site preference (SP) in N_2_O measured with quantum cascade laser absorption spectroscopy (QCLAS). In addition, we discuss the challenges of incorporating molecular biological techniques in this scheme.

## Biological pathways for NO and N_2_O production

**Figure 1:**
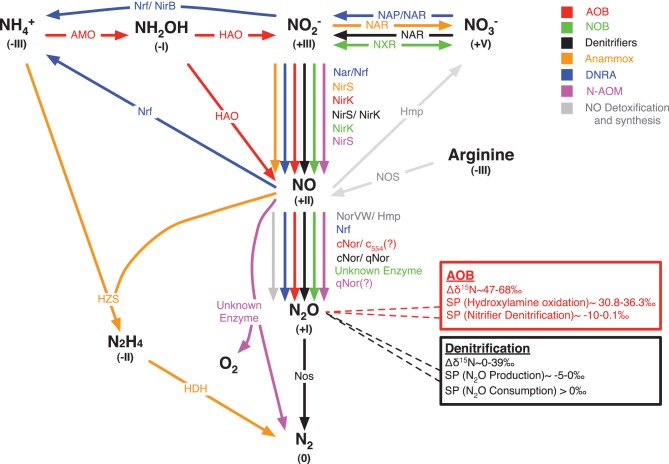
**Biological pathways for NO and N_2_O turnover in the catabolic branch of the N-cycle plus NO synthesis and detoxification.** Different colors are allocated to different microbial guilds or turnover pathways: AOB (red), ammonia oxidizing bacteria; NOB (green), nitrite oxidizing bacteria; anammox (orange), anaerobic oxidation of ammonia; DNRA (blue), dissimilatory nitrate/nitrite reduction to ammonia; N-AOM (purple), oxygenic nitrite-dependent anaerobic oxidation of methane. Key enzymes of each microbial guild are depicted that are known to mediate the conversion from one chemical N-species into another: AMO, ammonia monooxygenase; HAO, hydroxylamine oxidoreductase; NXR, nitrite oxidoreductase; Nar, membrane-bound nitrate reductase; Nap, periplasmic nitrate reductase; NirK, copper-containing nitrite reductase; NirS, cytochrome *cd*_1_ nitrite reductase; Nrf, cytochrome *c* nitrite reductase; NirB, cytoplasmic nitrite reductase; cNor, nitric oxide reductase that accepts electrons from c-type cytochromes; qNor, nitric oxide reductase that accepts electrons from quinols; c554, cytochrome *c*_554_; NorVW, flavorubredoxin, Hmp, flavohemoglobins; HZS, hydrazine synthase; HDH, hydrazine dehydrogenase; Nos, nitrous oxide reductase; NOS, nitric oxide synthase; unknown enzymes, nitric oxide dismutation to N_2_ and O_2_ during N-AOM and nitrous oxide producing enzyme in NOB. Roman numbers in brackets denote the oxidation state of the chemical N-species. The red and the black box denote the isotopic composition (δ^15^N) and the site preference (SP) in isotopomers of N_2_O produced by AOB and denitrifiers, respectively.

The study of laboratory cultures for pathways and controls of NO and N_2_O production in different organisms has generated considerable knowledge, which was partly reviewed recently (Stein, 2011; Chandran et al., 2011). Figure 1 shows that the sequential reduction of nitrite (NO^−^_2_) to NO and further to N_2_O can be performed by all organisms involved in the catabolic branch of the N-cycle. While all N-cycle organisms can perform these reactions it is currently believed that denitrifiers and ammonia oxidizing bacteria (AOB) and ammonia oxidizing archaea (AOA) are the most important environmental sources of N_2_O. However, in the following section we additionally review the evidence for NO and N_2_O production by nitrite oxidizing bacteria (NOB), anaerobic methane (N-AOM) and AOB (anammox), and bacteria that perform dissimilatory nitrate reduction to ammonia (DNRA). Even though it is clear that these bacteria can produce NO and N_2_O there is only few information on the controls, conditions and magnitude for NO and N_2_O production by these bacteria in the laboratory and in the environment. This should be an important aspect of future research as e.g., DNRA and anammox are the major N-conversion pathways in some important environments.

### Denitrification

The key enzyme for NO formation during denitrification is nitrite reductase (Nir). Purification and characterization of Nir from several bacteria revealed two entirely different periplasmic enzymes: a heme-containing cytochrome *cd*_1_ Nir (NirS) and a copper-containing Nir (NirK) as reviewed by Cutruzzolà (1999). Reduction of NO to N_2_O is mediated by respiratory nitric oxide reductases (Nor). Respiratory Nor proteins are integral membrane proteins that fall into two groups: one is a cytochrome *bc* complex that can use c-type cytochromes as electron donors (cNor), whereas the other one lacks a cytochrome *c* component and accepts electrons from quinols (qNor; sometimes termed NorZ) (Hendriks et al., 2000; Zumft, 2005). Few bacteria use qNor for classical denitrification. Rather, qNor is mainly encoded by pathogenic bacteria that use it for NO detoxification and the survival of anoxic periods when expressed in concert with Nir, as shown for *Neisseria spp*. (Anjum et al., 2002; Rock et al., 2007). The final step in denitrification is mediated by nitrous oxide reductase (Nos), a multi-copper enzyme that reduces N_2_O to dinitrogen (N_2_) (Zumft and Kroneck, 2007).

N_2_O reduction by Nos is the only known N_2_O consuming process that can counteract release of N_2_O from ecosystems (Richardson et al., 2009). Accumulation of N_2_O is often observed in pure cultures (Baumann et al., 1996; Otte et al., 1996; Kester et al., 1997; Bergaust et al., 2010) and mixed microbial communities (Firestone and Tiedje, 1979; Firestone et al., 1980; Morley et al., 2008; Kampschreur et al., 2008b; Schreiber et al., 2009; Elberling et al., 2010; Pellicer-Nàcher et al., 2010; Liengaard et al., 2011) during transitions from anoxic to oxic conditions or *vice versa* (Table 1). Even in pure cultures the physiological basis for this is not well understood because it probably has multiple, strain-specific reasons. It has been hypothesized that Nos is—unlike Nir and Nor—inhibited by O_2_ (Morley et al., 2008), but in pure cultures evidence for O_2_-insensitive (Berks et al., 1993) and O_2_-sensitive (Otte et al., 1996) Nos have been reported. Likewise, it has been argued that expression of Nos is slower than that of the preceding denitrification enzymes (Firestone et al., 1980; Stief et al., 2009), but in *Paracoccus denitrificans* Nos synthesis is faster (Baumann et al., 1996; Bergaust et al., 2010) and in *Pseudomonas stutzeri* Nos is even constitutively expressed at low levels (Körner and Zumft, 1989). More studies on Nos expression in relation to N_2_O production pathways and on Nos inhibition by O_2_ are needed with environmentally relevant isolates and mixed microbial communities. Additional factors that lead N_2_O accumulation are the slower turnover of Nos at low pH as compared to nitrate reductase (Nar), Nir, and Nor (Richardson et al., 2009; Bergaust et al., 2010), low pH during Nos assembly (Bergaust et al., 2010), inhibition of Nos by nitrous acid formed from NO^−^_2_ at low pH (Zhou et al., 2008), inhibition of Nos by exogenously produced NO (Frunzke and Zumft, 1986; Schreiber et al., unpublished results) or hydrogen sulfide (H_2_S) (Sørensen et al., 1980) and copper limitation (Granger and Ward, 2012).

**Table 1:** **Transient formation of NO and N_2_O in different habitats**.

**Habitat**	**Perturbation**	**NO [μM]**				**N_2_O (μM)**				**Possible pathway**	**Reference**
		**Baseline**	**Peak**	**Build-up^a^**	**Recovery^b^**	**Baseline**	**Peak**	**Build-up^a^**	**Recovery^b^**		
Tropical soil (slurries)	Oxic-anoxic	−	−	−	−	0	200–400	13–20 h	6–10 h	Denitrification	Liengaard et al., 2011
Agricultural soil (cores)	Oxic-anoxic by liquid-manure injection	−	−	−	−	<1	200	27 h	48 h	Denitrification	Markfoged et al., 2011
Agricultural soil (aggregates)	Oxic-anoxic by tryptone addition	−	−	−	−	<1	400	19.5 h	n.d.	Denitrification	Hojberg et al., 1994
Permafrost soil (cores)	Oxic-anoxic by thawing	−	−	−	−	<1	2.5	36 h	n.d.	Denitrification	Elberling et al., 2010
Nitrifying and denitrifying biofilm	Oxic-anoxic	<0.03	1.1	5–7 min	15 min	0.5	5	5 min	15 min	AOB	Schreiber et al., 2009
	Oxic-anoxic	<0.03	0.3	30 min	n.d.	<0.1	3	30 min	n.d.	Denitrification	
	NO^−^_2_ addition	<0.03	1.3	0.5 min	20 min					AOB	
	NO^−^_2_ addition	0.05	0.4	1 min	n.d.					Denitrification	
Full scale nitritation reactor	Influent shut-down	15 ppm^c^	80 ppm^c^	~10 min	1	10	110	4.5 h	n.d.	AOB/Denitrification and reduced gas stripping	Kampschreur et al., 2008a
Complex nitrifying culture	Oxic-anoxic	0.3 ppm^c^	2.5 ppm^c^	~ 8 min	n.d.	2	11	10 min	n.d.	AOB	Kampschreur et al., 2008b
	NO^−^_2_ addition	0.2 ppm^c^	0.45 ppm^c^	15 min	45 min	2.4	3.4	15 min	30 min	AOB	
Membrane-aerated biofilm	Oxic-anoxic	−	−	−	−	<1	70	25 min	60 min	AOB	Pellicer-Nàcher et al., 2010
	Anoxic-oxic	−	−	−	−	20	45	20 min	25 min	Denitrification	
Freshwater sediment	Salinity increase	−	−	−	−	0	4	9 h	22 h	Denitrification	Nielsen et al., 2009
Marine sediment	Salinity decrease NO^−^_3_ increase	−	−	−	−	0	2.5	2	7 h	Denitrification	
Arabian sea water	Oxic-anoxic		−	−	−	0.05	1.5	72 h	48 h	Denitrification/AOB	Naqvi et al., 2000

a Time to reach peak concentrations.

b Time to recover to a new steady-state concentration (not necessarily to baseline concentration).

c Concentration in ppm instead of μM because it was measured in the gas phase.

### Ammonia oxidizing bacteria (AOB)

High levels of NO and N_2_O can be produced by pure cultures of aerobic AOB (Lipschultz et al., 1981; Kester et al., 1997; Shaw et al., 2006), but the mechanism is not completely understood. Generally, two different pathways are inferred. First, the activity of nitrifier-encoded NirK and cNor reduces NO^−^_2_ to NO and N_2_O in a pathway termed nitrifier denitrification (Poth and Focht, 1985; Wrage et al., 2001; Schmidt et al., 2004b). A few reports exist on N_2_ formation by AOB during nitrifier denitrification, but a *nosZ* gene or functional Nos in AOB was not demonstrated (Poth, 1986; Schmidt et al., 2004b; Schmidt, 2009). The term nitrifier denitrification is somewhat misleading as it has until now not been shown that it is a true dissimilatory process for energy conservation and growth, but rather may be a detoxification mechanism to counteract the accumulation of NO^−^_2_ to toxic concentrations (Beaumont et al., 2002, 2004a,b).

In the second pathway, N_2_O is formed by hydroxylamine (NH_2_OH) oxidation. The current model is that hydroxylamine oxidoreductase (HAO) oxidizes NH_2_OH to NO (Hooper, 1968; Hooper and Terry, 1979). NO is then reduced to N_2_O by a yet unidentified Nor; a potential candidate is cytochrome *c*_554_ (Upadhyay et al., 2006). However, the catalytic cycle of HAO, including its intermediates and its catalytic potential are a subject of ongoing debate (Hendrich et al., 2002; Cabail and Pacheco, 2003; Cabail et al., 2005; Fernández et al., 2008; Kostera et al., 2008) and as of yet direct formation of N_2_O from HAO or other reactions can not be excluded. Indeed, the difference in the SP of N_2_O produced by NH_2_OH oxidation and nitrifier denitrification indicates that N_2_O might be produced by HAO by a mechanism that (1) either does not involve NO reduction by canonical Nor used for nitrifier denitrification or (2) does proceed via a completely different mechanism without free NO as intermediate (discussed in section “site preference” and “HNO as intermediate of enzymatic hydroxylamine oxidation”). Both nitrifier denitrification and NH_2_OH oxidation require O_2_ to activate ammonia (NH_3_) with ammonia monooxygenase (AMO) to NH_2_OH, which serves as a substrate for HAO or as electron donor to nitrifier denitrification. A pathway in which AOB perform denitrification with organic substrates instead of NH_3_ as electron donor (Schmidt, 2009) should be considered heterotrophic denitrification performed by AOB. AOA have also been demonstrated to produce N_2_O probably by pathways akin to AOB (Santoro et al., 2011).

The relative importance of NH_2_OH oxidation and nitrifier denitrification for NO and N_2_O production is still debated. Based on pure culture investigations Yu et al. (2010) hypothesized that a high NH_3_ oxidation activity favors N_2_O production via NH_2_OH oxidation. Similarly, Wunderlin et al. (2012) found that NH_2_OH oxidation is favored by high NH_3_ and low NO^−^_2_ concentrations, and a high nitrification rate in a mixed culture for treating municipal wastewater. Moreover, stable nitrogen isotopes work with AOB pure cultures showed that NH_2_OH oxidation contributes to N_2_O production mainly at high O_2_ whereas nitrifier denitrification is more active at low O_2_ concentrations (Sutka et al., 2006).

### Nitrite oxidizing bacteria (NOB)

NOB form NO and N_2_O during denitrification of nitrate (NO^−^_3_) or NO^−^_2_ with pyruvate or glycerol as electron donor under anoxic conditions (Freitag et al., 1987; Ahlers et al., 1990), but a known NO reductase could not be identified in the genomes of different Nitrobacter species and “*Candidatus* Nitrospira defluvii” (Starkenburg et al., 2006, 2008b; Lücker et al., 2010). Under anoxic conditions nitrite oxidoreductase (NXR) mediates NO^−^_3_ reduction to NO^−^_2_, while it mediates the reverse reaction under oxic conditions (Freitag et al., 1987). NOB actively express NirK, which co-purifies with NXR, in the presence of NO^−^_2_ and if O_2_ concentrations are low (Ahlers et al., 1990; Starkenburg et al., 2008a). NO generated by NOB-NirK is thought to direct cellular electron flux either toward O_2_ respiration at high O_2_ concentrations or toward NADH synthesis by reversibly inhibiting cytochrome oxidase at low O_2_ concentrations. An interesting question to explore in natural communities would be whether NO produced by AOB or denitrifying bacteria can influence the activity of NOB.

### Dissimilatory nitrate reduction to ammonia (DNRA)

NO and N_2_O turnover by bacteria that perform DNRA has been mainly investigated in *Escherichia coli* and *Salmonella typhimurium*. In *E. coli*, NO formation is mediated by cytochrome *c* nitrite reductase (Nrf) under anoxic conditions in the presence of NO^−^_3_ and NO^−^_2_ (Corker and Poole, 2003). NO detoxifying enzymes, such as flavorubredoxin, may further reduce NO to N_2_O. On the other hand, *E. coli* Nrf reduces NO to N_2_O or NH_3_ if electrons are donated to the enzyme at high or low potential, respectively (Costa et al., 1990), contributing to detoxification of exogenously generated NO (van Wonderen et al., 2008). Aerobic and anaerobic NO formation from NO^−^_2_ in *S. typhimurium* is mediated by membrane-bound nitrate reductase (Nar). Under aerobic conditions, activity of NO detoxifying Hmp (see below) oxidizes NO to NO^−^_3_ resulting in non-detectable NO concentrations in culture suspensions (Gilberthorpe and Poole, 2008).

### Anaerobic methane and ammonia oxidizing bacteria

Bacteria that mediate the oxygenic nitrite-dependent oxidation of methane (N-AOM) and anaerobic ammonia oxidation (anammox) have been shown to use NO as an intracellular intermediate produced by NO^−^_2_ reduction via NirS while they consume exogenous NO without concurrent N_2_O formation (Ettwig et al., 2010; Kartal et al., 2010, 2011). Rather, N-AOM dismutates NO to form N_2_ and O_2_, while anammox couples the reduction of NO to a condensation with NH_3_ to produce hydrazine (N_2_H_4_). Both have the genetic potential to reduce NO to N_2_O; anammox bacteria encode for flavorubredoxin (Strous et al., 2006) and N-AOM encodes for qNor (Ettwig et al., 2010). However, physiological data for both indicates that they withstand rather high NO levels (N-AOM 20 μmol L^−1^, anammox 7 μmol L^−1^) without activating anaerobic NO detoxification mechanisms.

### NO^−^_2_ → NO → N_2_O: central steps in the N-cycle

Generally, the reduction of NO^−^_2_ to NO is a central step in the catabolic branch of the N-cycle, because it can be carried out by all involved organisms (Figure 1). The reduction of NO^−^_2_ to NO is central for energy conservation in denitrification, anammox and N-AOM. In contrast, during NO^−^_2_ oxidation and nitrifier denitrification the reduction of NO^−^_2_ to NO is involved in regulating metabolic homeostasis or the removal of toxic NO^−^_2_ (Beaumont et al., 2002, 2004a; Starkenburg et al., 2008a).

The reduction of NO to N_2_O is, besides a potential direct formation of N_2_O from NH_2_OH in AOB, the only known biochemical reaction that produces N_2_O. NO reduction to N_2_O is central for energy conservation only in denitrification (Zumft, 1997). The function of cNor in AOB is unclear. cNor is expressed and metabolically active during aerobic growth (Beaumont et al., 2004b). Knock-out mutants of cNor have lower growth rate and yield in chemostats (Schmidt et al., 2004b), but not in batch culture (Beaumont et al., 2004b). In chemostats, cNor regulates the free NO concentration to an optimal, non-toxic level and contributes to recovery of AOB from anaerobic conditions (Schmidt et al., 2004b). On the other hand, stripping NO from AOB cultures leads to the inhibition of growth, arguing for NO being an obligate intermediate of AOB (Zart et al., 2000).

### NO detoxification and NO synthesis

Most bacteria encode for enzymes involved in NO detoxification. This is true for bacteria inside and outside the catabolic N-cycle. Flavohemoglobins (Hmp) mediate the O_2_-dependent detoxification of NO to NO^−^_3_ with NO dioxygenase activity (Gardner et al., 1998). In contrast, the anaerobic detoxification of NO is mediated by Flavodiiron NO reductase (flavorubredoxin [NorVW]) and Hmp by reducing NO to N_2_O (Kim et al., 1999; Gardner et al., 2002; Gomes et al., 2002).

An alternative, less explored route to N_2_O formation is via the synthesis of NO from arginine by NO synthases (NOS) and subsequent reduction of NO to N_2_O by cNor, qNor, Hmp or NorVW. Because NOS was discovered in the medical field it shares a similar abbreviation with N_2_O reductases (Nos). Until now, NOS has only been detected in a few bacterial –mostly gram-positive – species (Sudhamsu and Crane, 2009) and synthesized NO seems to remain intracellular (Shatalin et al., 2008; Schreiber et al., 2011). However, NOS activity has also been reported in blooming, pelagic diatoms (Vardi et al., 2006). More research is needed to elucidate if NOS-derived NO is a significant source for N_2_O emitted from phytoplankton blooms in oceans and freshwater.

## Chemical reactions in NO and N_2_O turnover

Chemical production of NO and N_2_O from inorganic nitrogen compounds at ambient temperatures are well known phenomena in soil science (van Cleemput and Samater, 1996) and atmospheric chemistry (Lammel and Cape, 1996). In soil science, the chemical processes leading to NO and N_2_O are often summarized as chemo-denitrification (Chalk and Smith, 1983). NH_2_OH and NO^−^_2_ (or its acid HNO_2_) are the main precursors for chemical production of NO and N_2_O in wastewater or natural waters. In the following, we discuss chemical reactions involving HNO, NH_2_OH, and NO^−^_2_ that can be responsible for the release of NO and N_2_O. We will also discuss the possible significance of chemical N_2_O production during biological NH_2_OH oxidation.

### Significance of HNO

In many studies on chemical N_2_O production, HNO is postulated as the direct precursor of N_2_O (see below): HNO dimerizes via hyponitrous acid (H_2_N_2_O_2_), to N_2_O and H_2_O (Bonner and Hughes, 1988).

(1) $$2\text{ HNO}\to {\text{H}}_{2}{\text{N}}_{2}{\text{O}}_{2}\to {\text{N}}_{2}\text{O}+{\text{H}}_{2}\text{O}$$

It can be assumed that formation of HNO in natural and wastewater follows the same mechanisms that are used to synthesize HNO (DuMond and King, 2011) in the laboratory: (1) disproportionation of NH_2_OH derivatives containing good leaving groups attached to the nitrogen atom, and (2) decomposition of nitroso compounds (X–N=O, where X represents a good leaving group). Chemical HNO production are likely to occur during wastewater treatment, since nitrification can produce considerable amounts of both, HNO_2_, which is a precursor for nitrosation agents (e.g., dinitrogen trioxide N_2_O_3_, Bonner and Stedman, 1996), and NH_2_OH.

Recently, medical researchers have started to reevaluate the relevance of HNO for physiologically and biologically systems (Fehling and Friedrichs, 2011). The increased interest in HNO is due to the fact that HNO lifetime in aqueous solutions is much longer than previously assumed: the HNO dimerization rate constant has been reassessed to be on the order of 8 × 10^5^ M^−1^ · s^−1^ instead of the previously reported value of 2 × 10^9^ M^−1^ · s^−1^, and the *pK*_*a*_ value of HNO has been redetermined to be 11.4 instead of the old value of 4.2 (Shafirovich and Lymar, 2002). It is likely that the importance of HNO has also been underestimated in the research on N_2_O emissions. Analytical determination of HNO is very challenging (Miranda, 2005), because HNO is short-lived. However, computer simulations could be a helpful tool to assess the importance of HNO in N_2_O formation (Law et al., 2012).

### HNO_2_ disproportionation

A well understood process for NO production is the disproportionation of HNO_2_ (Udert et al., 2005). Since the *pK*_*a*_ value of the NO^−^_2_/HNO_2_ couple (*pK*_*a*_ = 3.29; Schwartz and White, 1981) is far below 7, this process releases relevant amounts of NO only under acidic conditions. The disproportionation of HNO_2_ can be described with Equation 2. The products—NO and NO_2_—are in equilibrium with N_2_O_3_ (Equation 5) which is an important agent for nitrosation (Bonner and Stedman, 1996). Under aerobic conditions, NO will be further oxidized to NO_2_. Since NO_2_ reacts with H_2_O to form HNO_2_ and NO^−^_3_, the reaction scheme (Equations 2–4) is ultimately a chemical pathway for the oxidation of NO^−^_2_ to NO^−^_3_.

(2) $$\text{2 }{\text{HNO}}_{2}↔\text{NO}+{\text{NO}}_{2}+{\text{H}}_{2}\text{O}$$

(3) $$\text{NO}+0.5\;{\text{O}}_{2}\to {\text{NO}}_{2}$$

(4) $$2\;{\text{NO}}_{2}+{\text{H}}_{2}\text{O}↔{\text{HNO}}_{2}+{\text{NO}}_{3}^{-}+{\text{H}}^{+}$$

(5) $$\text{NO}+{\text{NO}}_{2}↔{\text{N}}_{2}{\text{O}}_{3}$$

Since the kinetic and equilibrium constants for Equations 2–5 are known, the production of NO can be calculated (Udert et al., 2005). Depending on the aeration intensity, substantial losses of nitrogen oxides can occur during chemical HNO_2_ oxidation. The stripped nitrogen oxides are mainly HNO_2_, but also NO is lost.

### Iron-mediated reduction of NO^−^_2_

Ferrous iron [Fe(II)] can reduce NO^−^_2_ to NO and, in the second reaction step, NO to N_2_O (Kampschreur et al., 2011).

(6) $$\begin{array}{c} {\text{NO}}_{2}{}^{-}+{\text{Fe}}^{2\text{+}}+2{\text{H}}^{+}\to {\text{Fe}}^{3\text{+}}+\text{NO}+{\text{H}}_{2}\text{O}\\ \;\Delta {\text{G}}_{0}=\text{35}.\text{8 kJ }{\text{reaction}}^{-1} \end{array}$$

(7) $$\begin{array}{c} \text{NO}+{\text{Fe}}^{\text{2+}}+{\text{H}}^{+}\to {\text{Fe}}^{3\text{+}}+\text{0}.\text{5 }{\text{N}}_{2}\text{O}+\text{0}.\text{5 }{\text{H}}_{2}\text{O}\\ \;\Delta {\text{G}}_{0}=-\text{38}.\text{9 kJ }{\text{reaction}}^{-1} \end{array}$$

The first reaction is thermodynamically not possible under standard conditions, but in natural waters ferric iron [Fe(III)] will precipitate and thereby draw the Gibbs free energy to negative values. Iron-mediated reduction of NO^−^_2_ was described as one of the sources of N_2_O in soils (van Cleemput, 1998). Recently, Kampschreur et al. (2011) postulated that this process can contribute significantly to N_2_O production in wastewater treatment, if NO^−^_2_ and Fe(II) are present concomitantly. One example for such a system is nitrogen removal from anaerobic digester effluents via nitritation/denitrification or nitritation/anammox. Digester supernatants can contain high amounts of Fe(II), because iron salts are used to precipitate phosphate and Fe(II) will be released in the anaerobic digester due to the reducing conditions. Hu et al. (2001) reported an additional reaction of NO^−^_2_ with iron: under acidic conditions NO^−^_2_ is reduced in the presence of metallic iron to N_2_ and NH_3_. They propose a mechanism, in which metallic iron is oxidized at low pH releasing Fe^2+^ ions and molecular hydrogen (H_2_). NO^−^_2_ is then reduced by H_2_ to N_2_ and NH_3_.

### Oxidation of NH_2_OH by FE(III)

Iron not only mediates NO and N_2_O production from NO^−^_2_. As Fe(III), it also oxidizes NH_2_OH to N_2_O. This process can be used for the analytical determination of trace amounts of NH_2_OH (Butler and Gordon, 1986a). The general equation for the reaction is

(8) $$4\text{ Fe}(\text{III})+2\;{\text{NH}}_{2}\text{OH}\to 4\text{ Fe}(\text{II})+{\text{N}}_{2}\text{O}+{\text{H}}_{2}\text{O}+4\;{\text{H}}^{+}$$

In this reaction, N_2_O formation strongly depends on the pH value. In experiments with distilled water and natural seawater, Butler and Gordon (1986b) found that at pH 3, N_2_O recovery was 80%, while at a pH value of 9.5, N_2_O production was negligibly low. The authors hypothesized that at high pH values, HNO, reacts with O_2_ to produce NO^−^_2_ and H_2_O. However, it is also known that HNO can react with NH_2_OH to N_2_ (Bonner et al., 1978, Equation 10). Chemical production of N_2_O via NH_2_OH oxidation by Fe(III) is a likely process during nitrification, because Fe(III) compounds are ubiquitous in natural waters and wastewater treatment systems.

### Reaction of NH_2_OH with HNO_2_ and HNO

Döring and Gehlen (1961) investigated the reaction of NH_2_OH and HNO_2_. They described the process as nitrosation of NH_2_OH. The overall reaction can be written as

(9) $${\text{NH}}_{2}\text{OH}+{\text{HNO}}_{2}\to {\text{N}}_{2}\text{O}+2\;{\text{H}}_{2}\text{O}$$

In their reaction scheme, Döring and Gehlen (1961) included H_2_N_2_O_2_ (the dimer of HNO) as a direct precursor for N_2_O. At neutral pH values, N_2_O_3_ is the relevant nitrosation agent. There are several reaction pathways for N_2_O_3_ formation from HNO_2_. Formation of N_2_O_3_ from HNO_2_ is given by Equations 2 and 5. A kinetic constant for nitrosation of NH_2_OH is given by Döring and Gehlen (1961) and together with the kinetic constants for Equations 1 and 4 (Udert et al., 2005) the N_2_O production from NH_2_OH and HNO_2_ can be estimated. Some of the NH_2_OH can also react with the intermediate HNO to form N_2_ (Bonner et al., 1978)

(10) $$\text{HNO}+{\text{NH}}_{2}\text{OH}\to {\text{N}}_{2}+2\;{\text{H}}_{2}\text{O}$$

### Disproportionation of NH_2_OH

The disproportionation of NH_2_OH can be described with the following equation (Bonner et al., 1978):

(11) $$4\;{\text{NH}}_{2}\text{OH}\to 2\;{\text{NH}}_{3}+{\text{N}}_{2}\text{O}+{\text{H}}_{2}\text{O}$$

In pure water, this process is very slow with slightly higher degradation rates at elevated pH values. At pH 3 and 25 ± 3°C, Bonner et al. (1978) observed no NH_2_OH disproportionation over 2 months, while 12–18% of the NH_2_OH was degraded over 2 months at pH 13.5. Complexes of transition metals can accelerate NH_2_OH disproportionation considerably (Alluisetti et al., 2004). Jenni et al. (2012) also observed N_2_O formation within minutes, although the experiment was conducted in a phosphate buffer solution without transition metals. The disproportionation might have been catalyzed by the steel surface of an electrode immersed in the reactor, but this hypothesis still has to be proven.

### Autoxidation of NH_2_OH

Oxidation of NH_2_OH with O_2_ (autoxidation, Equation 12) is a slow process, although faster than NH_2_OH disproportionation.

(12) $$2\;{\text{NH}}_{2}\text{OH}+{\text{O}}_{2}\to {\text{N}}_{2}\text{O}+3\;{\text{H}}_{2}\text{O}$$

Again, trace concentrations of metals can strongly accelerate the process. Anderson (1964) reported that in an aerated solution with 1 mmol·L^−1^ NH_2_OH and 1 μmol·L^−1^ cupric sulfate 30% of the NH_2_OH was oxidized within 1 h, while only 2.5% were degraded without cupric sulfate addition (pH between 7.8 and 7.9, 30°C). Cu is by far the most potent catalyzer for the autooxidation of NH_2_OH followed by Co(II), Fe(II), Mn(II), and Zn(II) (Moews and Audrieth, 1959). Since most wastewaters and natural waters contain some traces of metals, autoxidation of hydroxylamine cannot a priori be excluded as a source of N_2_O.

### HNO as intermediate of enzymatic NH_2_OH oxidation

Several authors postulated that HNO was a likely intermediate of HAO due to the observed N_2_O production (Anderson, 1964; Ritchie and Nicholas, 1972). Igarashi et al. (1997) could show that the crystal structure of HAO in *Nitrosomonas europaea* is in agreement with the following two step reaction

(13) $${\text{NH}}_{2}\text{OH}\to \left(\text{HNO}\right)+2\;{\text{H}}^{+}+2\;{\text{e}}^{-}$$

(14) $$\left(\text{HNO}\right)+{\text{H}}_{2}\text{O}\to {\text{HNO}}_{2}+2\;{\text{H}}^{+}+2\;{\text{e}}^{-}$$

Based on this scheme, an imbalance of the two reaction steps could lead to an accumulation of HNO and subsequently to chemical N_2_O production (Equation 1). Law et al. (2012) developed four different metabolic computer models to elucidate the mechanisms of aerobic N_2_O production in a nitritation reactor. The best fit of the measurement data was achieved with a model based on chemical HNO production. The other models, which represented three different metabolic pathways for the enzymatic reduction of nitrite and NO to N_2_O, could not reproduce the measurement data satisfactorily. Indeed, we think that the positive SP of N_2_O produced during NH_2_OH oxidation can be explained by a kinetic isotope effect acting during the chemical cleavage of a symmetric intermediate such as H_2_N_2_O_2_ formed by dimerization of two HNO molecules (Equation 1; Toyoda et al., 2005). In addition, the studies of Law et al. (2012) and of Udert et al. (2005) exemplify that computer models are powerful tools to elucidate the mechanisms of N_2_O and NO production, especially when the processes contain microbial as well as chemical reaction steps.

### Relevant environments for chemical reactions

In the last years, nitrogen treatment of high-strength wastewaters such as digester supernatant, manure and urine have received considerable attention. Based on our literature review, these systems are particularly prone to chemical production of NO and N_2_O because of high NH_3_ oxidation rates and high concentrations of the intermediate NH_2_OH. Furthermore, some treatment schemes include NO^−^_2_ accumulation as a process step, for example SHARON®. Ubiquitous iron compounds, e.g., from phosphate precipitation or as sensors and reactor walls, are another factor that can support the production of NO and N_2_O. At the current stage of knowledge, it is hard to estimate the contribution of chemical processes to the overall NO and N_2_O production. Many chemical processes have been described, but with the exception of HNO_2_ disproportionation and the reaction of HNO_2_ with NH_2_OH, the kinetic data are insufficient for a reliable prediction of the production rates. Chemical production of NO and N_2_O can also occur in natural environments, where high ammonia inputs meet low pH values such as strongly fertilized soils (van Cleemput and Samater, 1996) or poorly buffered lakes (Schuurkes and Mosello, 1988). Furthermore, chemical oxidation of NO and N_2_O is an important process in the atmosphere (Lammel and Cape, 1996).

## NO and N_2_O formation in natural environments

### Nitric oxide

NO production and consumption has been studied in soils. The studies used inhibition of nitrification with low concentrations of acetylene (~10 Pa) to distinguish between NO turnover by nitrification and denitrification, assuming that acetylene does not inhibit N_2_O reductase at these concentrations. O_2_ availability, as regulated by soil moisture content, is the main factor controlling the mechanisms of NO release (Bollmann and Conrad, 1998). While denitrification is the only process that releases NO under anoxic conditions, nitrification dominates NO release under oxic conditions with highest rates at low O_2_ concentrations. In addition, soil pH, NH^+^_4_, NO^−^_3_, NO^−^_2_, and respiration are important soil variables that affect NO turnover (Gödde and Conrad, 2000).

Measurements of NO in seawater are rare, because concentrations are low and turnover is fast due to its reactivity. However, Zafiriou et al. (1980) found that surface water of the central equatorial Pacific is a NO source to the atmosphere. Here, NO is formed by photolysis of NO^−^_2_ during daytime and reaches concentrations in the picomolar range (Zafiriou and True, 1979). Moreover, NO is formed by microbial processes in the O_2_ minimum zone of the eastern tropical North Pacific (Ward and Zafiriou, 1988). Here, maximum NO turnover and concentration coincide with low O_2_ concentrations (10–100 μmol L^−1^) and some nitrification activity overlying the O_2_ minimum zone. In contrast, NO turnover and concentrations are low in the core of the O_2_ minimum zone. The exact source of NO remained unidentified, but it was hypothesized that nitrifiers produce NO under reduced O_2_ concentrations and that denitrifiers establish rather low NO concentrations in the core of the O_2_ minimum zone. NO formation has been measured in marine sediments (Schreiber et al., 2008) and a more detailed study of NO turnover has been performed in freshwater sediments (Schreiber et al., unpublished results). Both studies will be discussed in the section focusing on microelectrodes.

### Nitrous oxide

Generally, N_2_O formation has been investigated to greater detail and in a wider variety of habitats as compared to NO, because it is an environmental impact is considered to be stronger than that of NO and its turnover is easier to measure due to its chemical stability. At present, anthropogenic N_2_O emissions account for ~40% of the global N_2_O emissions (Montzka et al., 2011). Current estimates state that ~50% of the anthropogenic N_2_O is emitted from soils (Stein and Yung, 2003), 10% from estuaries and freshwater habitats (Beaulieu et al., 2011) and 3.2% are emitted from wastewater treatment plants (WWTP) (Kampschreur et al., 2009). We caution that future adjustments to these estimates are likely, and that these averages do not capture the high variability in emissions from selected environments. Recent work has suggested that emissions from WWTPs in particular are highly variable and may in some cases be up to an order of magnitude greater than previous estimates (Ahn et al., 2010; Lotito et al., 2012). Soils and aquatic habitats exposed to intense agricultural activities are the largest sources due to high N-input through fertilization. Since mixed microbial communities in soils are the largest anthropogenic source for N_2_O, its formation has been intensively studied and was recently reviewed (Baggs, 2011). N_2_O formation in WWTP has been reviewed by Kampschreur et al. (2009).

The ocean is an important source of N_2_O accounting for ~30% of the natural N_2_O emission (Stein and Yung, 2003). Large areas of the ocean are thought to be in equilibrium with the atmosphere, but regions of O_2_ depletion are significant sources of N_2_O (Elkins et al., 1978). In O_2_ minimum zones, N_2_O is generally produced to concentrations in the nanomolar range as O_2_ reaches low concentrations (Yoshida et al., 1989; Naqvi et al., 2000; Farias et al., 2007; Nicholls et al., 2007). High N_2_O accumulation was observed in surface water of the Arabian Sea and explained with frequent, turbulence-induced aeration of suboxic surface water (Naqvi et al., 2000). Likewise, O_2_ fluctuations, induced by the El Nino-Southern oscillation, have been proposed to affect N_2_O emission from the O_2_ minimum zone of the eastern South Pacific (Farias et al., 2007). Furthermore, marine and freshwater sediments emit N_2_O (Meyer et al., 2008; Nielsen et al., 2009). NO and N_2_O formation in sediments will be discussed in more detail in the section focusing on microelectrodes.

The occurrence of animals such as earthworms (Horn et al., 2003) in soils and macrofauna in fresh -or seawater habitats (Stief et al., 2009; Heisterkamp et al., 2010) enhances the emission of N_2_O in response to anthropogenic N-input. These animals ingest denitrifying bacteria and stimulate their activity probably with delayed expression of N_2_O reduction leading to enhanced N_2_O emissions.

### Experimental approaches

In most investigated habitats NO and N_2_O formation has been attributed to the NH_2_OH pathway by AOB, nitrifier denitrification and heterotrophic denitrification. There are three approaches to determine the contribution of the different pathways:

1. Indirect inference of pathways by excluding the activity of all other possible pathways, which can be achieved by using inhibitors or by removing the substrate (Kampschreur et al., 2008b; Schreiber et al., 2009; Stief et al., 2009; Wunderlin et al., 2012).
2. Measuring the isotopic signature of N_2_O (^15^N natural abundance or SP) and comparing the data to values of pure cultures (Yoshida, 1988; Yoshida et al., 1989; Sutka et al., 2006; Well et al., 2006; Charpentier et al., 2007; Wunderlin et al., unpublished results).
3. Application of ^15^N isotopically-enriched substrates and mass spectrometric measurements of N_2_O (Bateman and Baggs, 2005; Baggs, 2008).

In complex systems all of these approaches suffer from the coupled nature of nitrification and denitrification. This especially applies to studies where bulk measurements have been done even though micro-environmental heterogeneities are expected; e.g., in aggregates in wastewater treatment systems or in soil particles. In addition, it has become clear that NO and N_2_O are dynamically produced in response to changing environmental conditions (Kampschreur et al., 2008b; Schreiber et al., 2009). Transient NO and N_2_O concentrations can be orders of magnitude higher than under steady state. Conventional mass spectrometric measurements do not allow measurements with high temporal and spatial resolution, making approach 2 and 3 inaccessible to microscale and dynamic analysis of NO and N_2_O.

## Novel analytical methods

In the following sections, we will discuss different analytical methods (microelectrodes, mass spectrometry, and QCLAS) that can be used to allocate NO and N_2_O production to certain pathways by using one of the three approaches outlined above. Combining these methods and thus the different approaches will lead to a more firm pathway allocation. Microelectrodes can measure with high temporal and spatial resolution and in combination with other microelectrodes (NH^+^_4_, NO^−^_3_, NO^−^_2_, and O_2_) approach 1 can be used to allocate source pathways. Further, QCLAS can measure the SP in N_2_O dynamically and can be used to allocate N_2_O production pathways with approach 2. In addition, we will discuss the potential for other techniques that measure the isotopic composition of N_2_O and molecular methods to aid the understanding of NO and N_2_O formation in complex environments.

## Microelectrodes to capture micro-environmental distribution and temporal dynamics of NO and N_2_O

### NO and N_2_O microelectrodes

Microelectrodes belong to the tool box of microbial ecologists since Revsbech et al. introduced an O_2_ microelectrode in the early 1980s (Revsbech et al., 1980). The first N_2_O microelectrode for microbial ecology (Revsbech et al., 1988) was a combined O_2_/N_2_O sensor where an O_2_-reducing gold cathode was placed in front of an N_2_O-reducing silver cathode (both polarized at −800 mV) to avoid the interference of O_2_ with N_2_O detection. These sensors where difficult to manufacture and had a short life-time. Thus, Andersen et al. (2001) introduced an improved O_2_-insensitive N_2_O microelectrode. Insensitivity to O_2_ is achieved by placing a reservoir filled with alkaline ascorbate solution for the chemical reduction of O_2_ in front of the N_2_O-reducing cathode, which is separated from the ascorbate reservoir with a gas permeable silicone membrane. These N_2_O microelectrodes have a sensitivity of ~0.5 μmol L^−1^ and a spatial resolution of ~60 μm.

Electrochemical NO sensors for the detection of NO in biological systems are available since the early 1990s (Shibuki, 1990). Amperometric sensing of NO is commonly achieved by the oxidation of NO at a working electrode polarized with 0.7–0.9 V vs. a reference electrode (Ag/AgCl or Calomel) leading to the following anodic reaction:

(15) $$\text{NO}+2\;{\text{H}}_{2}\text{O}-3\;{\text{e}}^{-}\to {\text{NO}}_{3}^{-}+4\;{\text{H}}^{+}$$

**Figure 2:**
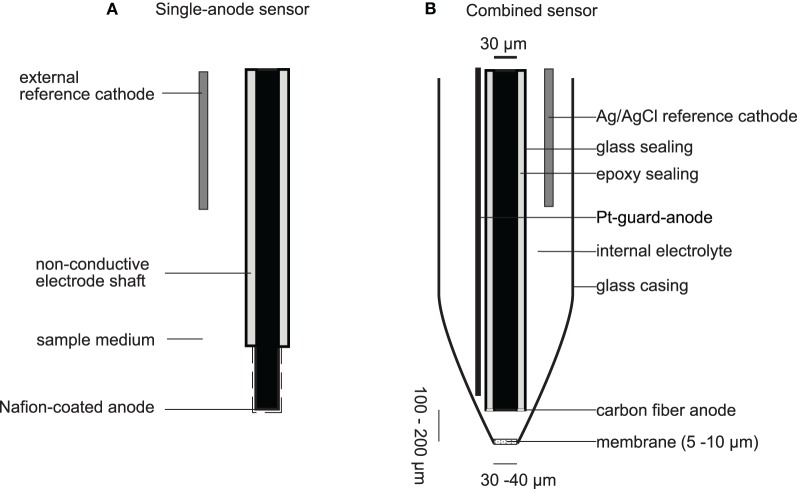
**NO microelectrodes. (A)** Depicts a typical single-anode type NO sensor with a long sensing anode, which is coated with Nafion to confer selectivity against charged interferences. The anode and reference cathode are directly emerged into the sample medium. Some sensor designs integrate the cathode into the electrode shaft. **(B)** Depicts the NO microelectrode for measurements in biofilms and sediments as reported by Schreiber et al. (2008). This sensor is also an example for a combined NO sensor (Clark-type) where sensing anode and reference cathode are separated from the sample medium by a gas permeable membrane. Drawing is not to scale.

The resulting current is proportional to the NO concentration and can be detected as the analytical signal. Electrodes are reported as single anode-type electrodes or as combined sensors (Figure 2). In combined sensors, the reference electrode and the sensing electrode are placed together in an internal electrolyte compartment that is separated from the sample by a gas permeable, non-conductive membrane (Clark-type, Figure 2B), whereas single anode-type electrodes use the aqueous sample as an electrolyte and complete the measuring circuit by submerging an external reference electrode into it (Figure 2A). Charged interferences like NO^−^_2_ and ascorbate are typically repelled by constructing combined sensors with hydrophobic membranes like chloroprene (Shibuki, 1990), PTFE (Teflon™) (Lee et al., 2004), sol-gels (Shin et al., 2005), polystyrene (Kitamura et al., 2000) or silicone (Schreiber et al., 2008), or by depositing conductive Nafion™ on single anode-type electrodes (Malinski and Taha, 1992; Friedemann et al., 1996; Bedioui and Villeneuve, 2003).

Most of the previously described NO electrodes have been optimized to detect NO at low nanomolar or even picomolar concentration. This has been achieved by increasing the sensing surface with a subsequent loss of spatial resolution. Single-anode-type sensors commonly rely on carbon-fibers that have a length of up to several millimeters and combined sensors have openings in the high micrometer to millimeter range. Microelectrodes with long, exposed sensing surfaces are not applicable for profiling in stratified microbial systems because the concentration of the analyte might change along the sensing surface. The obtained signal is then an integrated measure of the concentrations along the electrode. Similarly, combined electrodes with wide openings are also problematic for profiling applications, since the step size of different measurement points in a depth profile should not be smaller than two times the outer diameter of the electrode (Gieseke and de Beer, 2004). In addition, single-anode sensors are not robust enough to be inserted in a sturdy sediment or soil sample since the particles will damage the Nafion™ membrane that confers selectivity against NO^−^_2_. Consequently, applications of NO electrodes—commercially supplied, e.g., by World Precision Instruments (Sarasota, Florida, USA)—in microbiology were restricted to detection of NO in pure culture suspensions (e.g., Corker and Poole, 2003).

Recently, an NO microelectrode was introduced that is applicable to study complex, stratified microbial communities in sediments and biofilms (Schreiber et al., 2008). The NO microelectrode is a combined (Clark-type) sensor with a carbon-fiber anode (+750 mV) placed behind a gas permeable silicon membrane (Figure 2B). The sensor has a detection limit of 0.030 μmol L^−1^ and a spatial resolution of ~60 μm. Thus, the sensor is optimized to provide sufficient sensitivity for NO concentrations produced in complex, N-cycling microbial communities and sufficient spatial resolution to measure in microbial biofilms, sediments and soils. The robust Clark-type design allows measurements in sturdy soil and sediment samples. It has been made commercially available through Unisense A/S (Arhus, Denmark), who also supplies N_2_O microelectrodes.

### Interferences

H_2_S interferes with NO measurement as it passes the silicone membrane and is readily oxidized at the sensing anode. A sensitive H_2_S microsensor (Jeroschewski et al., 1996) should thus be used to rule out any interference of H_2_S in the measurements or –if possible- experiments must be designed to avoid active sulfate reduction in the sample by excluding sulfate from the medium. Jenni et al. (2012) investigated the interferences of CO_2_, O_2_, and various nitrogen compounds commonly found in wastewater treatment on NO and N_2_O sensors. They found that NO interfered with the N_2_O measurement, while the NO sensors were sensitive on NH_3_, NH_2_OH, HNO_2_, and N_2_H_4_. If high concentrations of these compounds are expected, it is recommended to check the concentrations of interfering compounds. No significant interferences were found by CO_2_ and O_2_. The cross-sensitivities can be corrected with calibration curves that are determined before the experiments. Jenni et al. (2012) also reported a significant temperature dependency. The NO signal increased by about 3.5% per 1°C and the N_2_O signal by 3.9% per 1°C. The temperature dependencies can be corrected with exponential functions.

### Application of NO microelectrodes

The novel NO microelectrode has been applied to study NO formation in permeable marine (Schreiber et al., 2008) and river (Schreiber et al., unpublished results) sediments. The results showed that in steady-state NO is produced in oxic/micro-oxic sediment strata reaching concentrations of 0.13 μmol L^−1^ in river and 0.5 μmol L^−1^ in marine sediments. In both sediments, NO produced in the oxic zone was consumed in the anoxic zone. It was hypothesized that NO was produced by AOB in the oxic zone. Labeling experiments with a ^15^N-labeled NO donor in the river sediment suggested that denitrification actively consumes exogenously produced NO.

Furthermore, the NO microelectrodes have been applied together with N_2_O microelectrodes in two N-cycling microbial biofilms; namely a complex NH^+^_4_-fed biofilm with nitrifying and denitrifying activity (Schreiber et al., 2009) and human dental plaque that was naturally exposed to high NO^−^_3_ and NO^−^_2_ in saliva (Schreiber et al., 2010). The study in dental plaque showed that plaque denitrified under aerobic conditions, that NO and N_2_O was produced by denitrification and that NO and N_2_O concentrations increased with decreasing pH. Aerobic denitrification has also been reported from permeable marine sediments (Gao et al., 2010) and from isolated (Patureau et al., 2000) or extracted soil bacteria (Morley et al., 2008). Until now, it is not known in which environments aerobic denitrification plays an important role, and if it is an environmentally significant NO and N_2_O emission pathway. NO, N_2_O, NO^−^_2_, NO^−^_3_, and O_2_ microelectrodes will be crucial to determine the importance of aerobic denitrification for NO and N_2_O release for complex ecosystems, because these sensors allow the simultaneous detection of NO, N_2_O, NO^−^_2_, NO^−^_3_, and O_2_ concentrations at high spatial resolution and their relation to denitrification activity.

Studying a complex N-cycling biofilm revealed the dynamics of NO and N_2_O formation upon perturbations in a system where nitrification and denitrification co-exist (Schreiber et al., 2009). The concomitant use of an O_2_ microelectrode and a set of control experiments enabled assignment of NO and N_2_O formation under oxic conditions to AOB and under anoxic conditions to denitrifiers. It also showed that AOB produce NO and N_2_O under fully oxic conditions if NO^−^_2_ concentrations are high. This is in agreement with other observations (Beaumont et al., 2004a,b; Shaw et al., 2006) and contradicts the assumption that that AOB *require* low O_2_ to release NO and N_2_O (Lipschultz et al., 1981; Poth and Focht, 1985; Kester et al., 1997; Beaumont et al., 2004a; Kampschreur et al., 2008b). The high temporal resolution of the microelectrodes allow to detect transient bursts (seconds to minutes) of NO and N_2_O. The bursts occurred by AOB upon O_2_ removal and upon NO^−^_2_ addition by both AOB and denitrifiers. The bursts only occurred if the perturbations were exerted upon metabolically active AOB and denitrifiers. In both scenarios NO and N_2_O are formed in parallel confirming that NO is the preceding intermediate of N_2_O in the N_2_O production pathways in this biofilm. An important contribution by Yu et al. (2010) showed that an AOB pure culture accumulated only NO, not N_2_O, upon transition from oxic to anoxic conditions. In mixed microbial communities were AOB and heterotrophic denitrifiers co-exist this could lead to NO release by AOB and immediate reduction to N_2_O by heterotrophic denitrifiers or anaerobic detoxification via NorVW and Hmp. This mixed source of N_2_O during transient oxic to anoxic conditions has to be taken into account when determining the pathways with isotopic techniques. It has been argued that N_2_O transiently accumulates during transition from anoxic to oxic conditions because O_2_ inhibits Nos while denitrification still proceeds, but direct evidence for this hypothesis is weak. Using both NO and N_2_O microelectrodes would allow to test this because N_2_O accumulation should not be accompanied by NO accumulation if the denitrification sequence is inhibited at the level of Nos.

### Application of N_2_O microelectrodes

In many habitats steady-state N_2_O concentrations are below or at the detection limit of the N_2_O microelectrode. Thus, the N_2_O microelectrode has commonly been used to estimate the denitrification potentials in stratified microbial communities such as sediments, biofilms, and aggregates in combination with the acetylene inhibition technique (Revsbech et al., 1988). Acetylene (~10 kPa) inhibits N_2_O reductase and leads to the accumulation of high amounts of N_2_O.

More recently, N_2_O microelectrodes have been used to study N_2_O production without acetylene inhibition in natural samples. These studies revealed that N_2_O concentrations in the micromolar range are expected when the system is exposed to a perturbation (Table 1). Transient accumulation of high N_2_O concentrations were achieved by any perturbation that affects the ambient O_2_ concentration: flooding of soils with water (Liengaard et al., 2011; Markfoged et al., 2011), creating an organic hotspot around a soil aggregate (Hojberg et al., 1994), thawing of permafrost soils (Elberling et al., 2010), and decreasing the O_2_ supply to wastewater-grown biofilms (Kampschreur et al., 2008a,b; Schreiber et al., 2009; Pellicer-Nàcher et al., 2010). In addition, increased input of NO^−^_3_, NO^−^_2_ or NH^+^_4_ to sediments, soils and biofilms (Hojberg et al., 1994; Meyer et al., 2008; Nielsen et al., 2009; Schreiber et al., 2009), organic inputs, salinity fluctuations in sediments (Nielsen et al., 2009) and changes of pH due to microbial activity in a denitrifying, dental biofilm (Schreiber et al., 2010) lead to increased micro-environmental N_2_O levels. Importantly, in many of these studies N_2_O accumulated in a transient manner making time-course measurements necessary to capture the N_2_O peak and the accumulation time span. The high spatial resolution of the N_2_O microelectrode allowed allocating processes that mitigate the emission of N_2_O to the atmosphere in soils, sediments and wastewater treatment biofilms. N_2_O that is produced by denitrification in deeper layers and is consumed during its diffusion toward the sediment-water interface in nutrient-enriched mangrove sediments (Meyer et al., 2008), toward the soil-atmosphere interface in a thawed permafrost soil (Elberling et al., 2010) or in a soil aggregate exposed to an organic hotspot (Hojberg et al., 1994). Likewise, N_2_O release from a membrane-aerated biofilm reactor was minimized by N_2_O-reducing microbes placed above AOB that produced N_2_O due to perturbations induced by an intermittent aeration regime (Pellicer-Nàcher et al., 2010).

### Outlook

From the investigations of transient NO and N_2_O accumulation it emerges that two scenarios with distinct dynamics are important. First, N_2_O accumulates over hours to days, because it mirrors the onset of denitrification activity. Depending on the system it decreases because N_2_O reduction pathways are turned on with a delay or denitrification activity decreases due to substrate limitation. Ahn et al. (2011) even observed that peak NO and N_2_O emissions after a shift to O_2_-limitation in a nitrifying reactor were lasting for several month before adaptation on the metabolic or community level decreased the emissions. Second, perturbation of active AOB or denitrifiers leads to burst-like (within seconds to minutes) release of NO and N_2_O. The exact biochemical mechanisms for this require further research directly on the involved enzymes. Moreover, future research must show the contributions of the two types of transitions to the N_2_O budget and could use this as a framework to mitigate peak N_2_O releases to the atmosphere. Mitigation strategies could aid at avoiding perturbations or confining the N_2_O-releasing processes into a diffusion-limited environment that is overlaid with N_2_O-consuming microbial communities.

## N_2_O source partitioning based on the nitrogen and oxygen isotopic signature

In recent years, the isotopic signature of N_2_O has been used as a powerful tool to assign N_2_O production pathways to AOB and heterotrophic denitrifiers in different ecosystems such as soils, rivers, sea, wastewater treatment (Yoshida et al., 1989; Yamagishi et al., 2007; Baggs, 2008; Koba et al., 2009; Baulch et al., 2011; Park et al., 2011; Toyoda et al., 2011). N_2_O is a linear molecule (N^β^-N^α^-O) with one nitrogen atom at the center position (N^α^) bound to oxygen, and one at the end position (N^β^) bound to N^α^. The three most abundant N_2_O isotopic species in the atmosphere are ^14^N^15^N^16^O (0.37%), ^15^N^14^N^16^O (0.37%) and ^14^N^14^N^16^O (>99%). Isotope abundances are usually reported in the δ-notation (in per-mil; ‰), δ^15^N = [(R_sample_/R_reference_)−1] × 1000, where R is the ratio of ^15^N/^14^N of a sample (R_sample_) with respect to atmospheric N_2_ as the reference (R_reference_) (Mariotti et al., 1981).

The intramolecular distribution of the nitrogen isotopes (^14^N^15^NO vs. ^15^N^14^NO) is termed SP and is expressed as the relative difference in δ^15^N between α and β position (SP = δ^15^N^α^–δ^15^N^β^) (Toyoda and Yoshida, 1999). In analogy to the δ-notation, the isotopomer analysis denotes the relative difference of the ^15^N/^14^N isotope ratio for a given position (δ^15^Nα, δ^15^N^β^) with respect to the standard {e.g., δ^15^N^α^ = [(^15^R^α^/^15^R^α^_reference_)-1] × 1000, whereas ^15^R^α^ = (^14^N^15^N^16^O)/(^14^N^14^N^16^O) and ^15^R^α^_reference_ is the isotope ratio of the standard material (N_2_O) (see below)} (Toyoda and Yoshida, 1999). The SP has the advantage of being independent of the isotopic signature of the respective substrates (e.g., NH^+^_4_ or NO^−^_3_) but of being specific for pathways (enzymes) involved in N_2_O formation (Toyoda et al., 2005; Sutka et al., 2006).

Microbial (enzymatic) processes usually lead to an isotopic fractionation due to different transformation rates of ^14^N and ^15^N, resulting in isotopically lighter end-products than molecules in prior steps (Stein and Yung, 2003). Thus, the average ^15^N/^14^N ratio of N_2_O, termed as δ^15^N^bulk^_N2O_, can be used to distinguish different production pathways in complex samples if the isotopic signature of the pure bacterial culture is known. However, the meaning of δ^15^N^bulk^_N2O_ can be limited since it is strongly-dependent on the isotopic signature of the substrate, which usually is unknown, as well as on the physiological activity (Mariotti et al., 1981). Additionally, the isotopic composition of an intermediate (e.g., N_2_O during heterotrophic denitrification) is affected by production (NO^−^_3_ reduction) as well as consumption (N_2_O reduction) processes.

In addition to nitrogen isotopes, oxygen isotope ratios are also increasingly used in order to better distinguish between the N_2_O formation pathways (Yoshinari and Wahlen, 1985; Kool et al., 2007; Baggs, 2008; Frame and Casciotti, 2010). In this case δ^18^O denotes the relative difference in the ^18^O/^16^O ratio of N_2_O (R_sample_) with respect to the reference (R_reference_), in per-mil (‰), usually being the Vienna Standard Mean Ocean Water (VSMOW) {δ^18^O = [(R_sample_/R_reference_)−1] × 1000} (Wahlen and Yoshinari, 1985).

## Analysis of the isotopic signature of N_2_O

There are basically two different analytical techniques available to analyze N_2_O nitrogen isotopic signatures at natural abundance levels (Table 2): (1) the isotope-ratio mass spectrometry (IRMS) (Brenninkmeijer and Röckmann, 1999; Toyoda and Yoshida, 1999), and (2) the recently developed QCLAS (Waechter et al., 2008).

**Table 2:** **Advantages and disadvantages of isotope-ratio mass spectrometry (IRMS), quantum cascade laser absorption spectroscopy (QCLAS) and membrane-inlet mass spectrometry (MIMS) adapted from Baggs (2008)**.

	**Advantages**	**Disadvantages**
IRMS	•Well known, widely applied method	•Lab-based method
	•Measurement of δ^15^N^α^, δ^15^N^β^ and δ^18^O	•Low temporal resolution (flask-sampling)
		•Requirement of standard gases (not commercially available)
QCLAS	•Portable, enabling field measurement campaigns	•Requirement of standard gases (not commercially available)
	•Continuous measurement (high temporal resolution) of δ^15^N^α^ and δ^15^N^β^	
MIMS	•High sample throughput	•Application limited to isotope labeling/tracer experiments
	•Low sample volume required	
	•Long-term measurement possible	
	•Online measurements with high temporal resolution possible	

### IRMS

IRMS-based method is widely applied with an excellent precision and accuracy (Mohn et al., 2010). Nevertheless, the calibration procedure of the intramolecular nitrogen isotope distribution in N_2_O is still under debate. Originally, two alternative approaches have been proposed, one by Toyoda and Yoshida (1999) and one by Brenninkmeijer and Röckmann (1999), which resulted in a difference in SP of about 30‰ for tropospheric N_2_O. The analysis of the SP by IRMS techniques relies on the N_2_O^+^ and NO^+^ fragment ions at the mass-to-charge ratio (*m/z*) 44, 45, 46 (for N_2_O) and *m/z* 30, 31 (for NO). However, both calibration approaches do not take into account the isotope effects associated with the formation of NO^+^ in the ion source of the mass spectrometer. Recently, Westley et al. (2007) investigated these discrepancies in more detail and found that these isotope effects have much smaller impact on the calibration procedure proposed by Toyoda and Yoshida (1999) (see below), and supported therefore this procedure as the most accurate basis for a community standard.

Furthermore, IRMS is a lab-based technique. Thus, the time resolution of N_2_O isotopic analysis during field measurement campaigns is therefore limited (Waechter et al., 2008). Nevertheless, in addition to nitrogen isotopes, the oxygen isotopic signature can also be analyzed routinely by IRMS.

### QCLAS

QCLAS is a novel approach for site-specific analysis of nitrogen isotopes, with the advantage of a high sensitivity, time resolution, and portability, the latter of which enables field measurement campaigns (Waechter et al., 2008). This was demonstrated by Mohn et al. (2012), who recently presented first data of a high precision real-time analysis of site-specific isotopic signatures of atmospheric N_2_O above a grassland plot. The measurement campaign was run over 3 weeks with almost 550 analyzed gas samples. It was demonstrated that a continuous measurement of the N_2_O isotopic signature allowed improved detection of the dynamics of N_2_O production (before and after fertilizer application to the grassland plot), and thus opens a completely new field of applications. In another study, isotopic signature of N_2_O, produced during batch-scale experiments with activated sludge, were analyzed in real time, which permitted to trace short-term fluctuations in SP and δ^15^N^bulk^_N2O_, allowing to identify N_2_O production pathways in biological wastewater treatment (Wunderlin et al., unpublished results).

The QCLAS is based on direct absorption laser spectroscopy in the mid-infrared range for simultaneous measurement of the most abundant N_2_O isotopic species, such as ^14^N^15^N^16^O, ^15^N^14^N^16^O, and ^14^N^14^N^16^O (Waechter et al., 2008; Mohn et al., 2010). In order to enable high precision analysis (e.g., a precision of <0.1‰ for δ^15^Nα and δ^15^Nβ) (Waechter et al., 2008) a combination with a pre-concentration unit is essential at ambient or sub-ambient mixing ratios (Mohn et al., 2010, 2012). For example, with the liquid nitrogen-free, fully-automated pre-concentration unit built by Mohn et al. (2010), N_2_O can be concentrated by a factor of 200 (e.g., from ambient concentrations to around 60 ppm) from 10 L gas samples within 20 min.

### Calibration

For both techniques, IRMS as well as QCLAS, an adequate calibration procedure needs to be applied, since instrumental nonlinearity and drifts impact the accuracy of the isotope ratio measurement (e.g., δ^15^N^bulk^_N2O_ values depend on the N_2_O gas concentration) (Waechter et al., 2008). However, international standards are not commercially available so far. Therefore, they need to be prepared and analyzed from other laboratories (intercalibration) for δ^15^N^bulk^_N2O_, δ^15^N^α^, and δ^15^N^β^, to ensure that measurements are performed on a common scale and that results are comparable between laboratories (Westley et al., 2007). So far, the calibration procedure proposed by Toyoda and Yoshida (1999), as mentioned above, is accepted as the provisional basis for a community standard: N_2_O is synthesized via thermal decomposition of isotopically characterized NH_4_NO_3_, since it is known that the nitrogen atom at the center (α) position of N_2_O originates from NO^−^_3_, while the end (β) nitrogen comes from NH^+^_4_. Using this calibration procedure a SP of tropospheric N_2_O of 18.7 ± 2.2‰ is measured (Westley et al., 2007).

### Membrane-inlet mass spectrometry (MIMS)

Membrane-inlet mass spectrometry (MIMS) was proposed as another promising tool to study the dynamics of N_2_O production in ^15^N labeling experiments. MIMS has a high sample throughput (within minutes), allows direct analysis of liquid or gas samples and requires only low sample amounts (Bauer, 1995; Baggs, 2008) (Table 2). Recently, it was coupled with an automated sampling and calibration unit (ASCU), and was tested in a long-term ^15^N-NO^−^_3_ tracer experiment over 7 days. It was confirmed that ^15^N measurements of N_2_ and N_2_O, detected as N_2_ at *m/z* 28, 29, and 30 (N_2_O was reduced to N_2_ in an elemental copper furnace prior to analysis), are in good agreement with IRMS-based analysis (Eschenbach and Well, 2011).

The membrane-inlet part can also be combined with a quadrupole mass spectrometer for simultaneous online measurement of different *m/z* ratios (e.g., ^15,15^N_2_O at *m*/*z* = 46, ^14,15^N_2_O at *m*/*z* = 45, ^15,15^N_2_ at *m*/*z* = 30, ^14,15^N_2_ at *m*/*z* = 29) with a time resolution of 1–2 min (Ettwig et al., 2010; Gao et al., 2010). Nevertheless, the interpretation of spectra corresponding to a certain gas mixture might be difficult since one peak can correspond to different atomic compositions (e.g., ^14,14^N^+^_2_ and CO^+^ at *m*/*z* = 28). This problem is reduced by applying ^15^N labeled substrates, where the only important remaining correction needed is for *m*/*z* = 30, which consist of the signal from the ^15,15^N^+^_2_ fragment of ^15,15^N_2_O, the ^14^NO^+^ fragment of ^14,14^N_2_O and ^15,15^N_2_) (Thomsen et al., 1994).

## Isotopic signature of N_2_O: site preference, δ^15^N and δ^18^O

### Site preference

The SP is a promising tool for N_2_O source partitioning since it is specific to pathways involved and independent of the respective substrates (Sutka et al., 2006) (Table 3). For N_2_O production via NH_2_OH oxidation by typical AOB pure cultures values in the range of 30.8 ± 5.9‰ to 35.6 ± 1.4‰ were measured (Sutka et al., 2003, 2004, 2006) which is in agreement with recently reported SP values of marine AOA (30.8 ± 4.4‰) (Santoro et al., 2011). In contrast, Frame and Casciotti (2010) estimated 36.3 ± 2.4‰ for a marine AOB. For nitrifier denitrification by AOB, the following SP values were reported: 0.1 ± 1.7‰ (Sutka et al., 2006), −0.8 ± 5.8‰ (Sutka et al., 2003, 2004) and −10.7 ± 2.9‰ (Frame and Casciotti, 2010). For N_2_O production via heterotrophic denitrification SP values in the range of −5.1‰ to 0‰ were reported (Toyoda et al., 2005; Sutka et al., 2006). Nitric oxide reductases (Nor) likely determine the SP of N_2_O during nitrifier denitrification as well as heterotrophic denitrification. The SP for both pathways is in the same range indicating that the involved Nor in AOB (cNor) and heterotrophic denitrifiers (cNor or qNor) (Stein and Yung, 2003; Stein, 2011) share a similar enzymatic mechanism. In case free NO is formed during NH_2_OH oxidation, any NO molecule that is funneled into nitrifier or heterotrophic denitrification (either directly or via initial oxidation to NO^−^_2_) would result in N_2_O with an SP of ~0‰ masking its initial NH_2_OH source.

**Table 3:** **Advantages and disadvantages of SP, δ^15^N^bulk^, and δ^18^O, on a natural abundance or labeled level [adapted from Baggs (2008)]**.

	**Advantages**	**Disadvantages**
Site preference (SP)	•Independent of isotopic signature of substrates	•Unknown pathways might affect SP
	•Noninvasive method	•SP from pure culture bacteria have to be known
	•Specific for pathways involved	
δ^15^N^bulk^	•Characteristic fractionation of different pathways (depending on the rate limiting step)	•Depending on the isotopic signature of the substrate, as well as the physiological activity
	•Noninvasive method	•Multiple reaction steps (branching effects) cause uncertainty
δ^18^O	•Noninvasive method	•Oxygen exchange between N species and O_2_ or H_2_O difficult to quantify
	•Additional information to nitrogen isotopic signature	
Isotope labeling of N and O	•Isotopically enriched substrates are not significantly impacted by kinetic isotope fractionation	•The use of ^18^O labeled H_2_O is not suitable under field conditions
	•Quantification of individual pathways	•Isotopically labeled substances might impact microbial activity

The most probable explanation for a positive SP during NH_2_OH oxidation is a preferable ^14^N-^16^O bond cleavage of a symmetric intermediate such as hyponitrite (^−16^O^14^N^15^N^16^O^−^), leading to an enrichment of ^14^N-^15^N-^16^O (Schmidt et al., 2004a; Toyoda et al., 2005). In the current model of N_2_O formation from NH_2_OH oxidation, NH_2_OH is reduced to NO, which is further reduced to N_2_O by an unidentified Nor. However, the positive SP of N_2_O formed from NH_2_OH oxidation can only be explained, (1) if the involved Nor has a different mechanism than Nor's mediating nitrifier and heterotrophic denitrification or (2) if N_2_O is formed by a different mechanism, which does not involve free NO. We suggest mechanisms involving HNO: either by formation of free H_2_N_2_O_2_ with further chemical decomposition to N_2_O (discussed in section “HNO as intermediate of enzymatic NH_2_OH oxidation”) or a site specific enzymatic cleavage of ^−^ONNO^−^ as discussed above (Schmidt et al., 2004a; Toyoda et al., 2005). Further insights in the enzymatic mechanism of HAO and potentially HAO-associated Nor with careful chemical control experiments are needed to elucidate the biochemical mechanism of N_2_O formation during NH_2_OH oxidation.

Furthermore, a positive SP is, in addition to NH_2_OH oxidation, also an indicator for increasing importance of N_2_O reductase activity relative to N_2_O production (substantially greater activity than 10% compared to production) (Yamagishi et al., 2007; Jinuntuya-Nortman et al., 2008; Koba et al., 2009). As a consequence, N_2_O reduction to N_2_ might lead to an overestimation of N_2_O production by NH_2_OH oxidation, or *vice versa*. Nevertheless, further investigations are necessary in order to determine the individual signatures under conditions more representative for ecosystems with mixed culture populations (Wunderlin et al., unpublished results).

Under nitrifying conditions, N_2_O can theoretically be produced simultaneously via NH_2_OH oxidation as well as nitrifier denitrification. Thus, based on SP literature data, the individual contribution (F_NN_: NH_2_OH oxidation; F_ND_: nitrifier denitrification) can be calculated from the following isotopomer mixing model:

(16) $${\text{F}}_{\text{ND}}=(\text{1}-{\text{F}}_{\text{NN}})=\frac{\left({\text{SP}}_{\text{tot}}{\text{-SP}}_{\text{NN}}\right)}{\left({\text{SP}}_{\text{ND}}{\text{-SP}}_{\text{NN}}\right)}$$

where SP_ND_ and SP_NN_ are the end-member SP signatures of the NH_2_OH oxidation and nitrifier denitrification pathway, respectively, as reviewed above, and SP_tot_ the measured signature of the individual produced N_2_O (Frame and Casciotti, 2010).

### δ^15^N

Wide ranges for δ^15^N^bulk^_N2O_ were reported so far, mainly due to limited information about the isotopic signature of the substrates or to both a huge complexity determined by multiple transformation processes involving different enzymes, as well as variable reaction rates or mechanisms affecting isotopic fractionation (Perez et al., 2006) (Table 3). For example, it was shown that isotopic fractionation during NH_3_ oxidation is variable, depending mainly on the amino acid sequences for the α-subunit of AMO of the different investigated pure culture AOB (Casciotti et al., 2003). However, N_2_O produced by AOB during nitrifier denitrification or NH_2_OH oxidation is basically more strongly depleted in ^15^N (Δ δ^15^N = δ^15^N_substrate_− δ^15^ N^bulk^_N2O_; in the range of between 40‰ and 68‰) compared to heterotrophic denitrification, where N_2_O is an obligate intermediate and the fractionation therefore depends on both production and consumption processes (Δ δ^15^N of 0–39‰) (Yoshida, 1988; Yoshida et al., 1989; Stein and Yung, 2003; Perez et al., 2006; Koba et al., 2009; Park et al., 2011).

### δ^18^O

The oxygen isotopic signature of N_2_O (δ^18^O) is also used as a tool for N_2_O source partitioning, even though this approach faces a couple of difficulties: for example, N_2_O production via NH_2_OH oxidation as well heterotrophic N_2_O reduction result in a positive correlation between the δ^18^O in N_2_O and SP (Frame and Casciotti, 2010) (Table 3). Furthermore, δ^18^O enrichment factors are scarce and highly variable (Park et al., 2011), and are reported to be strongly influenced by oxygen exchange or incorporation, such as (1) oxygen incorporation (from dissolved O_2_) into NH_2_OH during the oxidation of NH^+^_4_ to NH_2_OH, (2) oxygen incorporation (from H_2_O) into NO^−^_2_ during the oxidation of NH_2_OH to NO^−^_2_, and (3) oxygen exchange between NO^−^_2_/NO^−^_3_ and H_2_O (Kool et al., 2007). For example, it was shown that 64–94% of the oxygen atoms in the precursors of N_2_O were exchanged with oxygen atoms in H_2_O (Snider et al., 2009; Park et al., 2011), which underscores the fact that the understanding and quantification of the effect of oxygen exchange between H_2_O and dissolved nitrogen species is and will remain challenging. Isotopic labeling is a promising approach to overcome such difficulties (see below), but up to now the natural abundance oxygen isotopic signature should be used with caution in N_2_O source partitioning studies (Kool et al., 2007, 2010).

### N and O labeling

Beside natural abundances, nitrogen and oxygen isotope labeling techniques have been applied to study and quantify N_2_O production pathways (Table 3). For example, Poth and Focht (1985) investigated the relative importance of the NH_2_OH oxidation and nitrifier denitrification pathway in *Nitrosomonas europaea* pure culture by applying ^14^N-NH^+^_4_ in combination with ^15^N-NO^−^_2_. Based on the large amounts of double-labeled ^15,15^N_2_O (*m*/*z* = 46), it was concluded that nitrifier denitrification is the dominant pathway. Baggs and Blum (2004) determined the relative contribution of nitrification and denitrification to ^15^N-N_2_O production by the application of ^14^NH^15^_4_NO_3_ and ^15^NH^15^_4_NO_3_. However, such conventional ^15^N labeling techniques do not allow to distinguish between NH_2_OH oxidation and nitrifier denitrification in mixed population systems (Kool et al., 2010). As a consequence, a dual isotope approach was applied, based on ^18^O-labeling of H_2_O as well as ^15^N-labeling of NH^+^_4_ or NO^−^_3_ (Wrage et al., 2005). The basic concept behind is, that AOB use oxygen from O_2_ for the oxidation of NH^+^_4_ to NH_2_OH, but oxygen from H_2_O for the oxidation of NH_2_OH to NO^−^_2_ (see above). As such, the ^18^O signature of N_2_O produced via nitrifier denitrification reflect to 50% the signature of O_2_ and to the other 50% the signature of H_2_O, which is in this study artificially enriched in ^18^O (Kool et al., 2007), under the assumption that no further oxygen is exchanged between NO^−^_2_ and H_2_O. In contrast, the ^18^O signature of N_2_O derived from NH_2_OH oxidation reflects to 100% the signature of O_2_ (Wrage et al., 2005; Kool et al., 2010). Nevertheless, the effect of oxygen exchange has to be taken into account.

### Natural samples

The analysis of the natural abundance isotopic signature of N_2_O emitted from ecosystems such as soils, rivers or biological wastewater treatment indicate that N_2_O from terrestrial and aquatic sources is depleted in ^15^N compared to tropospheric N_2_O (δ^15^N = 7‰ and δ^18^O = 20.7‰) (Stein and Yung, 2003), but also show a huge variability and complexity, making process identification ambiguous at large scale. For example, in biological wastewater treatment an average δ^15^N^bulk^_N2O_ of −9.6‰, SP of 16‰ and δ^18^O of 22–44.3‰ were estimated (Yoshinari and Wahlen, 1985; Toyoda et al., 2011), indicating that nitrification as well as denitrification contributed to N_2_O production. N_2_O emitted from agricultural soils is reported to be strongly depleted in δ^15^N^bulk^_N2O_ (e.g., −34‰) (Park et al., 2011), referring to nitrification dominated N_2_O production. Isotopic signatures of N_2_O emitted from rivers and streams are in the range of −18‰ to 2.4‰ (δ^15^N^bulk^), −6‰ to 31‰ (SP) and 17‰ to 53‰ (δ^18^O) being in line with values reported above, which indicates to be highly influenced by sources such as agriculture or municipal wastewater treatment (Toyoda et al., 2009; Baulch et al., 2011). This is underscored by a recent study that investigates the oxygen and intramolecular nitrogen isotopic composition of N_2_O, confirming that nitrogen-based fertilizer application was largely responsible for the rise in N_2_O atmospheric concentration during the last 65 years (Park et al., 2012).

### Outlook

In this section, the isotopic signature of N_2_O, especially the SP, is discussed to be a powerful tool to distinguish N_2_O production pathways. Recent technological advances, e.g., the development and application of the QCLAS, now allow a high temporal resolution in the analysis of the isotopic changes of N_2_O. Nevertheless, an adequate calibration procedure still needs to be applied, since instrumental nonlinearity and drifts impact the accuracy of the isotope ratio measurement, and calibration standards are not commercially available so far. It is a pressing issue to further investigate the characteristic isotopic signatures of the individual N_2_O production pathways in mixed microbial communities under controlled conditions, in order to more accurately interpret isotopic signatures from complex environmental systems. Further, it is important to study N_2_O isotopic signatures with respect to involved microbial communities, enzymatic reaction mechanisms and enzymatic transformation rates. The use of the oxygen isotopic signature of N_2_O as a reliable tool for pathway identification requires the elucidation of mechanisms and rates of oxygen exchange in the future.

## Molecular approaches to understanding microbial no and N_2_O formation

While abiotic variables such as dissolved O_2_, pH, NO^−^_2_, and other nitrogen compounds have long been recognized to exert a strong influence on rates of microbial NO and N_2_O emissions, the importance of microbial community composition and dynamics to such emissions is still little understood (Wallenstein et al., 2006). As such, researchers have recently begun supplementing process-level NO and N_2_O emission measurements in a variety of environments with molecular techniques aimed at characterizing abundance, diversity, community structure, and activity of microbial guilds involved in nitrogen cycling. Here, we briefly introduce emerging molecular approaches to the delineation of key pathways, communities, and controls of NO and N_2_O production, and we summarize recent applications of these tools.

### Quantifying the genetic potential for N_2_O consumption

An appealing focus for application of molecular tools in environmental samples is direct quantification via the quantitative polymerase chain reaction (qPCR) of relevant functional genes (Smith and Osborn, 2008). Such an approach most commonly targets DNA, not RNA, and is thus a measure of genetic potential in the environment and not the activity.

Owing to the relative independence of each catabolic step, denitrification has been described as having a modular organization (Zumft, 1997). Indeed, Jones et al. (2008) concluded based on an analysis of 68 sequenced genomes of heterotrophic denitrifiers that approximately 1/3 lacked the *nosZ* gene encoding for N_2_O reductase and thus lack the genetic capacity for N_2_O reduction. Based on this assessment, researchers have hypothesized that the ratio of *nosZ* to the sum of *nirK* and *nirS* encoding for copper and cytochrome cd_1_-type nitrite reductases, respectively, is representative of the fraction of denitrifiers in a given environment that generate N_2_O as a catabolic end product. Environments with high *nosZ*/(*nirK* + *nirS*) ratios are likely associated with a high capacity for N_2_O consumption, and thus for low N_2_O emissions. Commonly used primers and qPCR conditions for genes relevant for NO and N_2_O turnover during N-cycling are available in the literature and are listed in Table 4, and thus the measurement of such ratios are feasible with little method development. Application of such tools has commonly shown a lower abundance of *nosZ* compared to other denitrifying reductases, particularly in soil environments (Henry et al., 2006; Hallin et al., 2009; Bru et al., 2011).

**Table 4:** **Reported primers and literature references relevant for NO and N_2_O turnover during N-cycling**.

**Target gene^a^**	**Primer name**	**Nucleotide sequence (5′–3′)**	**References**
b-AOB (amoA)	amoA-1F	GGG GTT TCT ACT GGT GGT	Rotthauwe et al., 1997
	amoA-2R	CCC CTC KGS AAA GCC TTC TTC	
AOA (amoA)	Arch-amoAF	STA ATG GTC TGG CTT AGA CG	Francis et al., 2005
	Arch-amoAR	GCG GCC ATC CAT CTG TAT GT	
narG	narG-F	TCG CCS ATY CCG GCS ATG TC	Bru et al., 2007
	narG-R	GAG TTG TAC CAG TCR GCS GAY TCS G	
napA	V17m	TGG ACV ATG GGY TTY AAY C	Bru et al., 2007
	napA4r	ACY TCR CGH GCV GTR CCR CA	
nirK	nirK1F	GGM ATG GTK CCS TGG CA	Braker et al., 1998, 2012
	nirK5R	GCC TCG ATC AGR TTR TGG	
	nirK876	ATY GGC GGV AYG GCG A	Henry et al., 2004
	nirK1040	GCC TCG ATC AGR TTR TGG TT	
nirS	nirS1F	CCT AYT GGC CGC CRC ART	Braker et al., 1998, 2012
	nirS6R	CGT TGA ACT TRC CGG T	
	cd3aF	GTS AAC GTS AAG GAR ACS GG	Michotey et al., 2000; Throbäck et al., 2004
	R3cd	GAS TTC GGR TGS GTC TTG A	
norB	cnorB-2F	GAC AAG NNN TAC TGG TGG T	Braker and Tiedje, 2003; Geets et al., 2007
	cnorB-6R	GAA NCC CCA NAC NCC NGC	
nosZ	nosZ2F	CGC RAC GGC AAS AAG GTS MSS GT	Henry et al., 2006
	nosZ2R	CAK RTG CAK SGC RTG GCA GAA	
	nosZF	CGC TGT TCI TCG ACA GYC AG	Kloos et al., 2001; Rich et al., 2003
	nosZR	ATG TGC AKI GCR TGG CAG AA	

a amoA – subunit A of ammonia monooxygenase, b-AOB - ammonia oxidizing bacteria, narG – subunit G of membrane bound nitrate reductase; napA – subunit A of periplasmic nitrate reductase; nirK - copper-type nitrite reductase; nirS - cytochrome cd_1_ nitrite reductase; norB – subunit B of nitric oxide reductase; nosZ – subunit Z of nitrous oxide reductase.

First assessments of this hypothesis are somewhat conflicting. In favor for the hypothesis, Philippot et al. (2009) demonstrated a negative correlation between *nosZ* proportional abundance and N_2_O/(N_2_ + N_2_O) ratio in grassland pasture soil. In a follow-up study, Philippot et al. (2011) dosed three soils with several dilutions of a denitrifying bacterial isolate known to lack the *nosZ* gene, and measured the response at the DNA level of *nirK*, *nirS*, and *nosZ* genes via qPCR. N_2_O emissions increased in all soils upon dosing of the *nosZ-*deficient isolate. However, in two of the three soils, the increase in denitrification potential (relative to non-inoculated controls) was higher than the measured increase in N_2_O emissions, suggesting that the original denitrifier community was capable of acting as a sink for N_2_O production. Moreover, ratios of N_2_O emissions to total denitrifying end products (N_2_O + N_2_) in non-inoculated soils were not correlated to *nosZ*/(*nirK* + *nirS*). While the authors acknowledge that abundance of *nosZ* deficient denitrifiers may not be as important in soils with a high N_2_O uptake capacity, their results clearly demonstrate that abundance of denitrifiers incapable of N_2_O reduction can influence denitrification end products in natural environments. Similarly, Morales et al. (2010) document a strong positive correlation between the difference in *nirS* and *nosZ* gene abundance (*nirS-nosZ; nirK* was not quantified) and N_2_O emissions in 10 soils. Garcia-Lledo et al. (2011) suggested that a significant decrease in *nosZ* gene abundance during periods of high NO^−^_3_ content in a constructed wetland might be indicative of increased genetic capacity for (unmeasured) N_2_O emissions.

In contrast, Čuhel et al. (2010) detail a significant but, puzzlingly, positive correlation in grassland soil between *nosZ*/(*nirS* + *nirK*) ratios and N_2_O/(N_2_+N_2_O), but caution that the relative importance of denitrifier community composition and enzyme regulation relative to proportion of *nosZ* deficient community members remains uncertain. In line with this result, Braker and Conrad (2011) found similar ratios of *nosZ*/(*nirS* + *nirK*) via Most Probable Number (MPN-) PCR in three soils with profoundly different N_2_O/(N_2_+N_2_O) ratios, and concluded that the hypothesis that a higher abundance of denitrifiers lacking *nosZ* is linked to increased N_2_O emissions may be an oversimplification.

The genetic potential for N_2_O production via nitrifier denitrification in AOB (and possibly AOA) could theoretically be measured via qPCR of the *nirK* and *norB* genes. Design of such analyses is hampered due to the fact that AOB *nirK* and *norB* genes are not phylogenetically distinct from that of heterotrophic denitrifying organisms (Cantera and Stein, 2007; Garbeva et al., 2007). In addition, NorB is not the only NO reductase in AOB (Stein, 2011).

### Community structure and diversity impacts on NO and N_2_O production

In addition to monitoring abundance of *nosZ* deficient denitrifiers, PCR-based tools are now being applied to the investigation of links between community structure and N_2_O emissions for both nitrifiers and denitrifiers. For this purpose, community structure is commonly profiled via cultivation-independent molecular fingerprinting methods, such as terminal restriction fragment length polymorphism (T-RFLP) or denaturing gradient gel electrophoresis (DGGE), targeting either 16S rRNA fragments specific to the functional guild of interest or functional genes (for example, *nirK* or *amoA*) directly. In addition, traditional cloning and Sanger sequencing and, increasingly, barcoded amplicon-based pyrosequencing of functional genes are often employed for robust phylogenetic comparisons. Readers are referred to Prosser et al. (2010) for a detailed methodological description of these and other nucleic-acid based methods. Multivariate statistical analyses such as canonical correspondence analysis (CCA), redundancy analysis (RDA) (Ramette, 2007; Wells et al., 2009), or path analysis (Avrahami and Bohannan, 2009) can then be used to explore the interplay between abiotic variables, community composition, and extant process rates.

It should be emphasized that the molecular and statistical tools highlighted above are most commonly used in microbial ecology to explore correlations, rather than causal associations, between community structure and function in complex microbial communities. As discussed in detail by Reed and Martiny (2007) directly testing causal relationships between microbial community composition or diversity and ecosystem processes is significantly more difficult, but experimental approaches often drawn from classical ecology are now being adapted to this challenge. We anticipate that future studies testing the functional significance of microbial community structure to NO or N_2_O production will benefit greatly from these approaches.

Studies targeting the relationship between nitrifier community composition and greenhouse gas production are sparse at present, despite the fact that ample molecular tools are available for this purpose. Avrahami and Bohannan (2009) employed a combination of qPCR and T-RFLP to explore the response of N_2_O emission rates and betaproteobacterial AOB abundance and composition in a California meadow to manipulations in temperature, soil moisture, and fertilizer concentration. While a complex interaction between factors was determined to directly and indirectly contribute to N_2_O emission rates, path analysis suggested that the major path by which NH^+^_4_ influenced emission rates in the high N fertilization treatment was indirectly via two specific AOB clusters. This observation suggested a significant relationship between AOB community structure and N_2_O emission rates. It is important to note that this study did not attempt to discriminate between the nitrifier denitrification and NH_2_OH oxidation pathways for AOB-linked N_2_O production, nor was the relative importance of heterotrophic denitrification vs. nitrification for overall N_2_O emissions directly compared.

Assessment of the importance of DNRA as a process, and diversity therein, to NO and N_2_O production is in its infancy. It has been suggested that our understanding of this little understood phenomena would benefit from the future investigations employing molecular techniques to quantify abundance and diversity of the *nrf* gene in conjunction with either modeling or stable isotope-based methods (Baggs, 2011). To our knowledge, such an assessment has yet to be conducted.

The relationship between denitrifier community composition and N_2_O emissions, while still ambiguous, has been studied in more detail. Palmer et al. (2010) investigated *narG* (encoding for membrane-bound nitrate reductase, Nar) and *nosZ* phylogenetic diversity in a low-pH fen via gene clone libraries and T-RFLP. They documented novel *narG* and *nosZ* genotypes and a phylogenetically diverse low-pH adapted denitrifier community, and suggested that the novel community structure may be responsible for complete denitrification and low N_2_O emissions under *in situ* conditions. In a more recent study, Palmer et al. (2012) investigated denitrifier gene diversity in peat circles in the arctic tundra via barcoded amplicon pyrosequencing of *narG*, *nirK/nirS*, and *nosZ*, and found evidence that high and low N_2_O emission patterns were associated with contrasting denitrifier community composition. Braker et al. (2012) found that, of three soils profiled, the soil with the most robust denitrification (lowest N_2_O/N_2_ ratio) harbored the most diverse denitrifier community, as measured via *nosZ* and *nirK* sequence diversity, suggesting that differences in community composition (higher diversity) are associated with ecosystem-level functional differences. In denitrifying bioreactors, population dynamics tracked via 16S rRNA-based T-RFLP were strongly correlated to NO^−^_2_ appearance and emissions of N_2_O (Gentile et al., 2007). In contrast, Rich and Myrold (2004) found little relationship between *nosZ* phylogenetic diversity as measured via T-RFLP in wet soils and creek sediments in an agrosystem, and suggested that activity and community composition were uncoupled in this ecosystem.

Taken together, the body of literature reviewed here suggests that, in at least some cases, community structure and diversity can play a functionally significant role in microbial N_2_O emissions. The importance of community composition relative to environmental parameters and metabolic adaptation in response to transient conditions (for example, shifts in patterns of gene expression or regulation) in determining N_2_O production, however, remains poorly understood. A worthwhile, but challenging future research direction would be to tease apart the influence of whole community metabolic adaptation versus community shifts on NO/N_2_O emissions in mixed microbial communities.

### A role for variation in regulatory response

Differences in transcriptional and translational regulation as well as enzyme activity have also been highlighted as potentially critical modulators of microbial NO or N_2_O production (Richardson et al., 2009; Bergaust et al., 2011; Braker and Conrad, 2011). Such differences likely contribute to observed associations between community structure and greenhouse gas production discussed above. Strong regulation at the transcriptional, translational, and enzyme level is likely occurring in both nitrifier and denitrifier communities, and such regulation complicates attempts to directly relate abundance or diversity of functional guilds to process rates (Braker and Conrad, 2011). Similarly, transient near-instantaneous NO and N_2_O accumulation in active nitrifying and denitrifying biofilms in response to O_2_ or NO^−^_2_ perturbations, as measured with high temporal resolution via microelectrodes, strongly suggests that dynamics are controlled in some cases at the enzyme level (Schreiber et al., 2009). Indeed, culture-based assays targeting denitrifier isolates from two soils demonstrated substantial diversity in sensitivity of Nos enzymes to O_2_ and provided a physiological underpinning for a previously observed link between denitrifier community composition and rate of N_2_O production (Cavigelli and Robertson, 2000).

Gene expression can be readily quantified with reverse transcriptase quantitative PCR (RT-qPCR), and researchers are now beginning to explore the relationship between gene expression patterns for critical functional genes (*amoA*, *hao*, *nirK*, *nirS*, *norB*, and *nosZ*) and NO/N_2_O emissions. Yu et al. (2010) used such an approach to quantify expression of *amoA*, *hao*, *nirK*, and *norB* in chemostats of *Nitrosomonas europaea* during initiation and recovery from transient anoxic conditions. Surprisingly, expression profiles of *nirK* and *norB* were not strongly linked; strong overexpression of *nirK* concomitant with NO accumulation was observed upon initiation of anoxia, and at the same time *norB*, *amoA*, and *hao* gene transcripts declined in abundance. N_2_O emissions peaked during recovery to aerated conditions, but did not correlate strongly to gene expression. The methods of Yu et al. (2010) provide a robust road map for examining relationships between nitrifier gene expression and NO/N_2_O emissions in mixed communities in environmental settings, though it should be noted that such an analysis is complicated by the polyphyletic nature of the AOB *nirK* and *norB* genes.

RT-qPCR has also been used to assess the relationship between gene expression and NO/N_2_O production in systems dominated by denitrifiers. Liu et al. (2010) quantified the relationship between *nirS*, *nirK*, and *nosZ* gene pools, their transcription products, and gas kinetics (NO, N_2_O, and N_2_) as a function of pH in soils. Interestingly, neither gene pool abundance, nor transcription rates could explain a profound increase in N_2_O emissions at low pH. The authors attribute the observed N_2_O:N_2_ product ratio to post-transcriptional phenomenon, although it is also plausible that enhanced chemo-denitrification may play a role.

A worthy future contribution could be made via direct environmental metatranscriptomic assessment of patterns in microbial gene expression in environments with different or varying rates of NO or N_2_O production. Metatranscriptomics is the direct sequencing of cDNA generated via reverse transcription of environmental RNA transcripts, and therefore provides a picture of currently transcribed genes in a given environment (Morales and Holben, 2011). In line with the results of Liu et al. (2010), it is important to recognize that measurement of the size or diversity of the gene transcript pool neglects post-transcriptional regulation governing, for example, the assembly of N_2_O reductase and enzyme activity (Braker and Conrad, 2011). As of yet, variations in post-transcriptional regulation at the community level and its effect on NO/N_2_O production has been little explored in nitrifying and denitrifying pure cultures and communities. Critical insights in this regard may be possible in the future from an approach coupling metatranscriptomics and metaproteomics—that is, direct measurement of the composition of the proteome in an environment.

## A need for an integrated approach to NO and N_2_O turnover in complex microbial communities

NO and N_2_O can be produced by many different biological and chemical reactions. Considerable progress has been made to allocate NO and N_2_O production to certain biological pathways, but commonly some uncertainty remains, because many processes share the same reaction sequence for N_2_O production via NO and NO^−^_2_. We delineated basically three-independent approaches to allocate pathways (indirect inference; isotopic signature of N_2_O, and isotopic labeling). Parallel use of these approaches will increase confidence in the interpretation. The possibility for various chemical reaction that produce and consume NO and N_2_O additionally complicate the picture. Chemical reactions can be important in engineered systems that employ waters with concentrated N-contents and in natural systems, where low pH values coincide with high ammonia inputs. However, in most natural systems and in municipal wastewater treatment, chemical reactions will probably not be the main contributors of NO and N_2_O emissions. Nevertheless, the possibility of chemical NO and N_2_O production has to be considered when interpreting measurements results. Experiments with inactivated biomass could help to give a first estimation of the chemical production rates. However, care has to be taken since the chemical conditions that facilitate chemical NO and N_2_O production such as pH and trace metal availability are in turn shaped by microbial activity.

Molecular methods have largely been applied independently from the stable isotope and microelectrode approaches. Ample opportunities exist for integration of these techniques. Indeed, it is clear that such an integrated approach is critical to assessing the importance of microscale heterogeneity in environmental parameters, microbial community structure and stability, and genetic regulation to observed process-level N_2_O emission rates.

Joint use of stable isotope methods in conjunction with molecular techniques appears particularly important, given reported difference in isotope effects depending on the community structure of nitrifiers (Casciotti et al., 2003) or denitrifiers (Toyoda et al., 2005) present. In addition, linking source-partitioned N_2_O as measured via stable isotope techniques to the underlying microbial communities via molecular approaches may allow a more significant measure of the strength of coupling between microbial diversity and measured emissions (Baggs, 2008, 2011). One promising way forward is to assess environmental conditions that favor a shift of dominant N_2_O production pathway (for example, from denitrification to nitrification, or *vice versa*) as measured via stable isotope methods, and to simultaneously link such a shift to diversity and abundance of functional gene pools and transcripts via PCR-based molecular approaches. Such an approach has the potential to yield insights into the relative importance of dominant functional guilds, community composition, and activity in determining microbial NO/N_2_O production rates. A fruitful first application would be to combine stable isotope-based methods with the molecular approach pioneered by Yu et al. (2010) for delineating the relationship between transcriptional response of the model AOB *Nitrosomonas europaea* and NO/N_2_O production. This coupled approach would allow conclusive verification of conditions proposed by Chandran et al. (2011) to favor a switch between nitrifier denitrification and NH_2_OH oxidation as dominant sources NO and N_2_O production.

Similarly, it is clear that molecular tools and microelectrodes are complementary to study NO and N_2_O turnover. An excellent example of such integration is provided by Okabe et al. (2011), who profiled microscale gradients in N_2_O emissions in anammox granules and compared these profiles to spatial location of AOB, as measured via fluorescence *in situ* hybridization (FISH). Based on their results, the authors concluded that putative heterotrophic denitrifiers in the inner part of the granule, not AOB, were likely responsible for the majority of the extant N_2_O process emissions. A similar approach is likely applicable in a wide variety of environments, including flocs, sediments, soils, and microbial mats. In addition, use of either FISH probes with higher phylogenetic resolution or depth stratified DNA/RNA extraction coupled to PCR-based measurements may allow a direct microscale assessment of links between microbial diversity and activity and NO/N_2_O production profiles. Such a microscale assessment is important because stratified environments likely contain both regions of N_2_O production and consumption that are masked during bulk NO/N_2_O concentration measurements or DNA/RNA extractions. In addition, microelectrode measurements with high temporal resolution should be combined with qPCR to better understand the regulation of NO and N_2_O peak emissions from different environments.

The conditions for NO and N_2_O formation in pure cultures and by chemical reactions begin to be better understood. Furthermore, several recent technological advancements allow researcher to investigate the regulation of NO and N_2_O formation in complex environments at high spatial and temporal resolution. These advancements provide a cornerstone to understand and mitigate the release of NO and N_2_O from natural and engineered environments.

### Conflict of interest statement

Frank Schreiber has a license agreement with Unisense A/S for the constrution and distribution of an NO microelectrode. The other authors declare that the research was conducted in the absence of any commercial or financial relationships that could be construed as a potential conflict of interest.
